# Minimally invasive adrenalectomy: a comprehensive systematic review and network meta-analysis of phase II/III randomized clinical controlled trials

**DOI:** 10.1007/s00423-022-02431-w

**Published:** 2022-01-12

**Authors:** Laura Alberici, Carlo Ingaldi, Claudio Ricci, Saverio Selva, Guido Di Dalmazi, Valentina Vicennati, Uberto Pagotto, Riccardo Casadei, Francesco Minni

**Affiliations:** 1 Division of Pancreatic Surgery, IRCCS Azienda Ospedaliero-Universitaria Di Bologna, via Albertoni 15, Bologna, Italia; 2 Department of Internal Medicine and Surgery, DIMEC) Alma Mater Studiorum, University of Bologna, S.Orsola-Malpighi Hospital, Bologna, Italy; 3 Dipartimento Di Scienze Mediche E Chirurgiche (DIMEC), Chirurgia Generale-Minni, Alma Mater Studiorum-Università Di Bologna, IRCCS, Policlinico S.Orsola-Malpighi, Via Massarenti n.9 40138, Bologna, Italy; 4 Unit of Endocrinology and Diabetes Prevention and Care, IRCCS Azienda Ospedaliero-Universitaria Di Bologna, via Albertoni 15, Bologna, Italia

**Keywords:** Laparoscopic adrenalectomy, Retroperitoneal adrenalectomy, Network meta-analysis, Safety, Efficacy

## Abstract

**Purpose:**

The best approach for minimally invasive adrenalectomy is still under debate.

**Methods:**

A systematic search of randomized clinical trials was carried out. A frequentist random-effects network meta-analysis was made reporting the surface under the cumulative ranking (SUCRA). The primary endpoint regarded both in-hospital mortality and morbidity. The secondary endpoints were operative time (OP), blood loss (BL), length of stay (LOS), conversion, incisional hernia, and disease recurrence rate**.**

**Results:**

Eight studies were included, involving 359 patients clustered as follows: 175 (48.7%) in the TPLA arm; 55 (15.3%) in the RPLA arm; 10 (2.8%) in the Ro-TPLA arm; 25 (7%) in the TPAA arm; 20 (5.6%) in the SILS-LA arm; and 74 (20.6%) in the RPA arm. The RPLA had the highest probability of being the safest approach (SUCRA 69.6%), followed by RPA (SUCRA 63.0%). TPAA, Ro-TPLA, SILS-LA, and TPLA have similar probability of being safe (SUCRA values 45.2%, 43.4%, 43.0%, and 38.5%, respectively). Analysis of the secondary endpoints confirmed the superiority of RPA regarding OP, BL, LOS, and incisional hernia rate.

**Conclusions:**

The best choice for patients with adrenal masses candidate for minimally invasive surgery seems to be RPA. An alternative could be RPLA. The remaining approaches could have some specific advantages but do not represent the first minimally invasive choice.

**Supplementary Information:**

The online version contains supplementary material available at 10.1007/s00423-022-02431-w.

## Introduction

Over the last 30 years, the minimally invasive approach to adrenalectomy has almost replaced open surgery in the management of most adrenal pathologies and tumors. Since 1992, year in which for the first time in literature Gagner [1] reported three cases of transperitoneal laparoscopic adrenalectomy performed by lateral approach with the patient in lateral decubitus, other surgical minimally invasive approaches to the adrenal glands had been introduced. One of these is the transperitoneal laparoscopic adrenalectomy performed by anterior approach with the patient in supine position, as reported by Lezoche et al [2] in a large series of cases in the late 1990s. Completely different approaches are the posterior retroperitoneoscopic adrenalectomy, with the patient in prone position, first described by Mercan [3], but later advanced and popularized by Walz [4], and the retroperitoneoscopic adrenalectomy with the patient in lateral decubitus, reported by Bonjer [5]. More recent minimally invasive techniques include the single-incision adrenalectomy for both laparoscopic or retroperitoneoscopic adrenalectomy and the robotic approach. Gaining experience in one or the other technique, many surgeons reported their cases in retrospective observational studies, sometimes testing their learning curve, other times comparing their results with those obtained with different techniques. All minimally invasive approaches have demonstrated their feasibility and safety. However, according to trials published so far, there is no consensus regarding which approach is the most suitable or has significant advantages over the others. Only a few meta-analyses are available, but data coming from prospective trials and comparative studies retrospective in nature are often mixed with each other creating concern about the accuracy and reliability of the analysis itself. Remarkably, any of the meta-analyses currently published in the literature reviewed only randomized controlled trials, which represent studies with the highest level of evidence. Moreover, meta-analyses constitute an important source of evidence-based practice but do have a significant drawback: they can compare only 2 interventions simultaneously.

Therefore, we chose to perform a network meta-analysis (NMA). This statistical evaluation preserves the randomized nature of data and has the advantage of assessing the effectiveness of several different approaches through the combination of both direct (head-to-head) and indirect (through common comparators) evidence to gain certainty about all treatment comparisons.

Our study aimed to define the safety and efficacy of the different approaches to adrenalectomy.

## Methods

A systematic review was performed according to the Cochrane recommendations [6], and the paper was structured following the PRISMA checklist (Preferred Reporting Items for Systematic Reviews and Meta-Analyses) [7]. The approval by an institutional review board was not required.

### Eligibility criteria

The eligibility criteria were established according to PICOS criteria [8]:

1. "Population": the "Population" was represented by the patients diagnosed with resectable benign or malignant adrenal tumor candidates to minimally invasive adrenalectomy (MIA)
2. "Intervention": the "Intervention" arms were all minimally invasive approaches different from transperitoneal laparoscopic lateral adrenalectomy
3. "Control": the "Control" group was the transperitoneal laparoscopic lateral adrenalectomy
4. "Outcomes": all studies reporting at least the postoperative morbidity and length of postoperative stay (LOS) were included
5. "Studies": all phase II/III RCTs, including at least two arms

NMA approach was used to avoid the problem derived from a multi-arm setting [9, 10]. The intervention arms were clustered according to three main parameters: the type of approach (transperitoneal or retroperitoneal), the surgical access (anterior, lateral, or posterior), and the application of particular devices for minimally invasive surgery (single incision or robotic approach). Thus, six intervention arms were planned: transperitoneal laparoscopic lateral adrenalectomy (TPLA); retroperitoneal minimally invasive lateral adrenalectomy (RPLA); transperitoneal laparoscopic lateral adrenalectomy with robotic approach (Ro-TPLA); transperitoneal laparoscopic anterior adrenalectomy (TPAA); single-incision laparoscopic adrenalectomy (SILS-LA); retroperitoneal minimally invasive posterior adrenalectomy (RPA). The TPLA was used as a referent arm. No other technical differences were used to additionally divide the surgical procedures to avoid an excessive scattering of the network.

### Information source, search, study selection, and data collection process

**Fig. 1:**
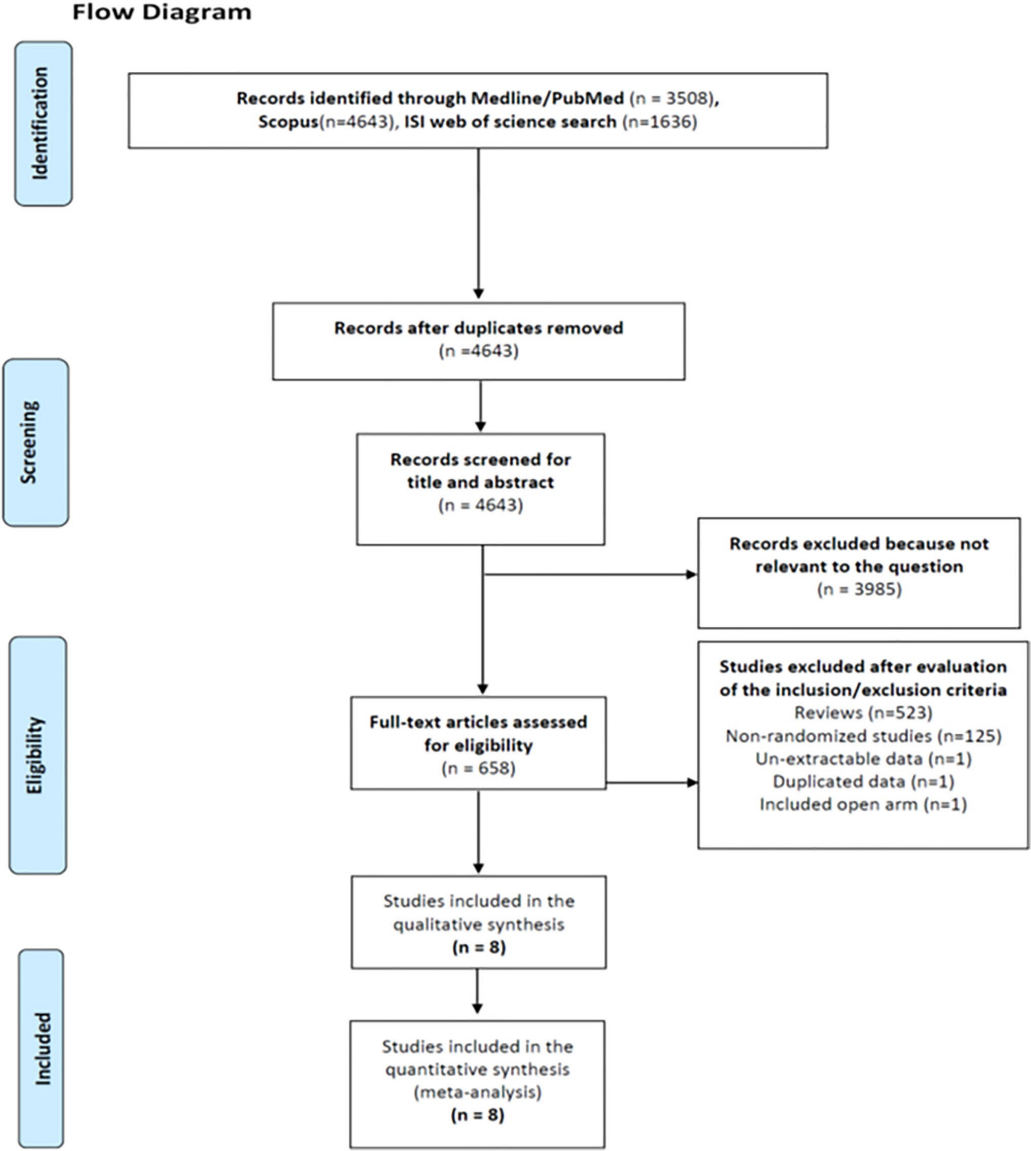
PRISMA flowchart. PRISMA = Preferred Reporting Items for Systematic Reviews and Meta-Analyses

The information sources were MEDLINE (via PubMed), Web of Science, and Scopus, and the last search was carried out on August 7, 2021. The studies' bibliography was included for additional reference. All eligible studies were read in full-text form by two independent investigators (G.D. and L.A.). When the inclusion criteria were fulfilled in the absence of exclusion criteria, the paper was included. Two independent reviewers (C.R. and C.I.) carried out data extraction using a dedicated form. Any disagreement between the reviewers was solved by a collegial discussion with the senior author (F.M.). A PRISMA flowchart was created to show the authors' conclusions (Fig. 1). Additional details regarding the eligibility criteria, information sources, search, study selection, and data collection process are exhaustively reported in a Supplementary file (S_Method).

### Data items

The primary endpoint was a composite endpoint, including the analysis of postoperative mortality and complications. The choice of using a composite endpoint was due to the low mortality rate that characterized the adrenal surgery, hence the need to group together mortality and complications as indicators of safety. The secondary endpoints were the analysis of operative time, blood loss, length of stay (LOS), conversion rate, incisional hernia, and disease recurrence rate. A safety/efficacy ratio was obtained using the composite endpoint (safety indicator) and LOS (efficacy indicator), respectively. Further details regarding the definition used for the primary and secondary outcomes are reported in a Supplementary file (S_Method).

### Geometries of the network and risk of bias within the individual study

The network geometries were obtained using nodes and edges to represent arms and trials, respectively. The nodes' sizes and the thicknesses of the edges in network graphs typically represent the amounts of respective evidence for specific nodes and comparisons [7]. Each network geometry was evaluated to recognize the presence of the common nodes. The absence of a common node precluded the analysis in the network for the arms. For all the endpoints, the contribution of each direct and mixed comparison was summarized using a matrix. The columns and rows indicated the direct and network estimates, respectively. The risk of bias within the individual studies was evaluated using a revised tool to assess randomized trials' bias (RoB2, latest version on July 9, 2019) [11].

### Summary measurements and methods of the analysis

The treatment effect was described as the surface under the cumulative ranking (SUCRA) curves and mean ranks. These values were calculated, starting from relative rank probability. The SUCRA value represents the odds, without uncertainty, that each intervention would be the best option. The mean rank represents the treatment position in a ranking in which the ideal treatment has a mean rank equal to 1 [12, 13]. Moreover, the SUCRA values of the primary endpoint and the LOS were plotted in a safety/efficacy diagram to assess the best choice [14]. Besides, the network estimates were also reported in a pairwise form (head to head comparison) using odds ratios (ORs) or standardized mean differences (SMDs) for dichotomous outcomes and continuous variables, respectively. The measurements were expressed using 95% confidence intervals (CIs) and 95% predicting intervals (PrIs). The network meta-analysis was based on the frequentist approach, and the ranking probabilities were obtained by drawing the coefficients 1000 times. The linear predictor for each study was evaluated for each draw, and the largest linear predictor was noted [15].

### Inconsistency, risk of bias across the studies, and meta-regression analysis

The network's reliability was assessed by evaluating the inconsistency [16] with local and global approaches. The local approach was measured within the closed loop and reported as the ratio of two odds ratios (RoR) or the absolute difference between the direct and indirect estimation (IF) with a 95% confidence interval. When a closed loop was absent, the inconsistency was evaluated with the global approach, using the Chi-square. The heterogeneity was measured and reported as tau (τ) [17]. When the τ value was > 0.5, a multivariate meta-regression analysis was made to identify the factors having a significant effect (*P* value < 0.05). The covariates analyzed were country, design (single-institution or multicenter study), mean age, body mass index (BMI), tumor size, rate of malignant tumors, phaeochromocytomas, side of adrenalectomy, type of healthcare system, and quality of the study. Publication/reporting bias was tested using Egger's and Begg's test [18] (*P* value < 0.05). When a publication bias was discovered, a “trim and fill” adjustment was made [19].

## Results

### Studies selected

The results of the systematic research of the literature following the PRISMA statement are reported in Fig. 1. The search identified 9787 records (3508 from PubMed, 4643 from Scopus, and 1636 from ISI Web of Science). Five thousand one hundred forty-four papers were excluded because they were duplicate publications according to the title. Of the remaining 4643 references, 3985 were excluded since, according to the title and abstract, they were not pertinent to our field of study. Six hundred fifty-eight full-text articles were reviewed. Of these, 649 were excluded: 522 were reviews, 125 were non-randomized studies, one was a randomized study without extractable data, one contained duplicated data, and one included patients with open approach. Finally, eight studies [20–27] were eligible for the analysis. On reviewing the data extraction, there was 100% agreement between the two reviewers. The characteristics of the selected studies are summarized in Table 1.

**Table 1:** Characteristics of the nine included studies

First Author/year	Affiliation/Country	Total patients randomized	Design	Diseases	Exclusion criteria	Healthcare system	QoL with Rob2	Outcomes
Fernandez-Cruz et al. 1996 [20]	Department of Surgery and Anesthesiology, Hospital Clinic, University of Barcelona, Barcelona, Spain	21	TPLA vs. RPLA	Benign unilateral or bilateral adenoma with Cushing disease	Other pathologies different from Cushing	NHS	Some concerns	a,b,c,d,e,f,g
Morino et al. 2004 [21]	Department of Surgery, Minimally Invasive Surgery Center, University of Turin, Italy	20	TPLA vs. Ro-TPLA	Benign unilateral functioning and non-functioning adrenal lesions	Tumor’s size > 10 cm; bilateral lesions; malignancy	NHS	Some concerns	a,b,d,e
Rubinstein et al. 2011 [22]	Section of Laparoscopic and Robotic Surgery, Glickman Urological Institute, Cleveland Clinic Foundation, Cleveland, Ohio	57	TPLA vs. RPLA	Benign and malignant mono-lateral functioning and non-functioning adrenal lesions	Age > 80 years; BMI > 40 kg/m^2^; bilateral adrenalectomy; prior abdominal surgery	VI	Some concerns	a,b,c,d,e,f,g
Lezoche et al. 2009 [23]	Department of Surgery ‘‘Paride Stefanini,’’ II Clinica Chirurgica, University ‘‘La Sapienza,’’ Roma, Italy	50	TPLA vs. TPAA	Benign unilateral functioning and non-functioning adrenal lesions	BMI > 35 kg/m^2^; tumor’s size > 8 cm; bilateral or right adrenalectomy; malignancy; ASA 4	NHS	Some concerns	a,b,d,e,f,g
Vidal et al. 2012 [24]	General and Endocrine Surgery Unit, Hospital Cĺınic i Provincial, Universitat de Barcelona, Spain	40	TPLA vs. SILS-LA	Benign unilateral functioning and non-functioning adrenal lesions	BMI > 39 kg/m^2^, tumor size > 4 cm, bilateral lesions; concomitant other procedures; pheochromocytoma	NHS	Some concerns	a,b,d,e
Mohammadi-Fallah et al. 2013 [25]	Urology, Nephrology, and Kidney Transplant Research Center, Imam Medical Center, Urmia University of Medical Sciences, Urmia, Iran	24	TPLA vs. RPLA	Benign and malignant unilateral functioning and non-functioning adrenal lesions	BMI > 40 kg/m^2^; prior major abdominal surgery; malignancy; tumor size > 6 cm; bilateral adrenalectomy;	NHS	Some concerns	a,b,c,d,e,f,g
Barczynski et al. 2014 [26]	Third Chair and Department of General Surgery, Jagiellonian University, Medical College, Krakow, Poland	61	TPLA vs. RPA	Benign mono-lateral functioning and non-functioning adrenal lesions	Major abdominal surgery; bilateral adrenal surgery; size > 7 cm Malignancy; pregnancy or lactation; age < 18 or > 80 years	NHS	Low	a,b,c,d,e,f,g
Chai et al. 2019 [27]	Seoul National University Hospital & College of Medicine, Seoul, Korea	83	TPLA vs. RPA	Benign mono-lateral functioning and non-functioning adrenal lesions	BMI > 35 kg/m2, age < 18 and age > 80, tumor size > 7 cm; no history of abdominal surgery	SI	Low	a,b,c,d,e,f,g

^ = Phaeochromocytomas; TPLA = transperitoneal laparoscopic adrenalectomy with lateral approach; RPLA = retroperitoneal mini-invasive adrenalectomy with lateral approach; Ro-TPLA = transperitoneal robotic adrenalectomy with lateral approach; OA = open adrenalectomy; TPAA = transperitoneal laparoscopic adrenalectomy with anterior approach; SILS-LA = single-port laparoscopic adrenalectomy with lateral approach; RPA = retroperitoneal mini-invasive adrenalectomy with the posterior approach; NHS = National Health System; VI = Voluntary Insurance; SI = Social Insurance; a = morbidity and mortality rate; b = operative time; c = blood loss; d = postoperative hospital stay; d = conversion to the open surgery; e = incisional hernia; f = recurrence of the disease;

### Study characteristics, network structures, and geometries

**Fig. 2:**
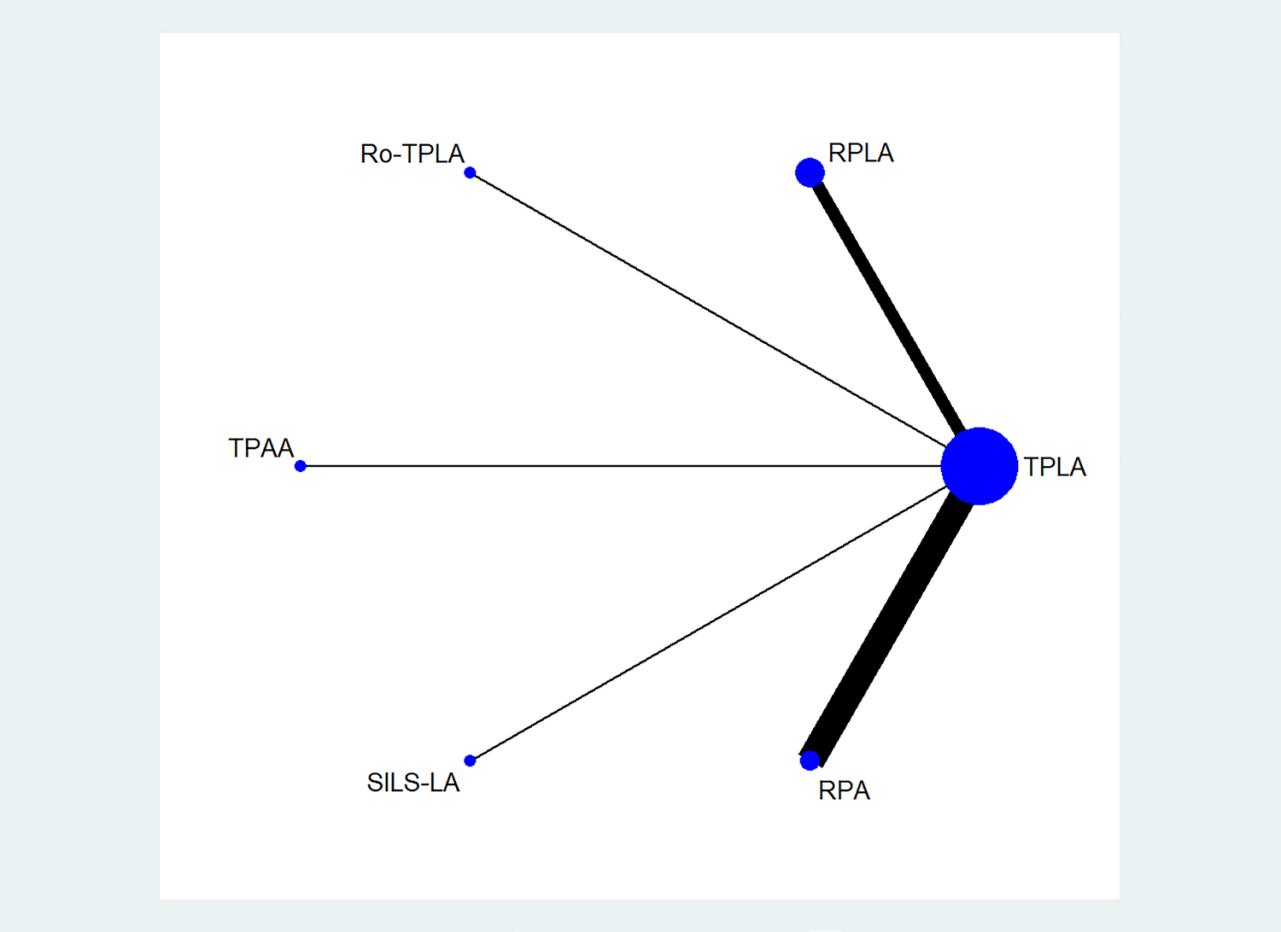
Network geometry for the primary endpoint (mortality or major complications). The network geometry graphically describes the direct comparisons available in the literature. In the figure, the blue nodes represent the interventions compared, while the edges represent the direct comparisons available (comparisons evaluated in at least one study) between pairs of interventions. TPLA = Transperitoneal laparoscopic lateral adrenalectomy; RPLA = retroperitoneal mini-invasive lateral adrenalectomy; Ro-TPLA = transperitoneal laparoscopic lateral adrenalectomy with robotic approach; TPAA = transperitoneal laparoscopic anterior adrenalectomy (TPAA); SILS-LA = single-incision laparoscopic adrenalectomy; RPA = retroperitoneal mini-invasive posterior adrenalectomy

Eight studies involving a total of 359 patients with adrenal lesions were included. The meta-analytical population had a mean age of 50 ± 10 years, a male–female ratio of 0.4. The mean size of the lesions was 3.4 ± 0.9 cm. The distribution of the interventions was the following: 175 (48.7%) in the TPLA arm; 55 (15.3%) in the RPLA arm; 10 (2.8%) in the Ro-TPLA arm; 25 (7%) in the TPAA arm; 20 (5.6%) in the SILS-LA arm; and 74 (20.6%) in the RPA arm (Supplementary Table 1). The primary endpoint's network geometry is shown in Fig. 2; there were six arms, five direct comparisons, only one common node (TPLA), and no quadratic or triangular loops. Several direct comparisons were lacking because all studies included the TPLA arm. Supplementary Fig. 1- panel A reports the contribution of each comparison. It should be noted that all direct comparisons contributed similarly to the network estimates. The operative time, LOS, and the conversion rate have a network geometry similar to the primary endpoint. On the contrary, blood loss, incisional hernia, and recurrence rate were reported in supplementary Figs. 2- panel A and B. No closed loops were found for the secondary endpoints. An exhaustive representation of contribution plots for all secondary endpoints is reported in Supplementary Figs. 2 (Panel B-G).

### Risk of bias within studies

The quality of the included studies is summarized in Table 1 and is detailedly reported in Supplementary Fig. 3: six studies [20–25] have "some concerns" in the randomization process, two studies [20, 21] in the "intended intervention," and six [20–25] in the "reported result." No study with a high risk of bias was included, but seven studies have some concerns in the overall evaluation. The matching for age, gender, tumor size, BMI, and right adrenalectomy is reported in Supplementary Table 2.

### Synthesis of results

The SUCRA and the mean rank values for the available intervention arm are shown in Table 2**,** while the relative ranking probabilities are displayed in Supplementary Table 3. The "head to head" comparisons for all endpoints are reported in Supplementary Fig. 4 (panel A-G).

**Table 2:** Surface under cumulative ranking area (SUCRA) values and mean rank for all outcomes. The SUCRA values express the percentage of each approach's safety or efficacy relative to an imaginary approach, which was always the best without uncertainty

Outcomes of interest	Studies	SUCRA (%) and Rank (mean) for arm											
		TPLA		RPLA		Ro-TPLA		TPAA		SILS-LA		RPA	
		SUCRA	Rank	SUCRA	Rank	SUCRA	Rank	SUCRA	Rank	SUCRA	Rank	SUCRA	Rank
Composite endpoint^	8	38.5	4.2	69.6	2.5	43.4	3.8	45.2	3.7	43.0	3.9	63.0	2.9
Operative time	8	45.1	3.7	45.1	3.7	35.7	4.2	54.5	3.3	32.1	4.4	87.5	1.6
Blood loss	5	28.6	2.4	26.6	2.5	*	*	*	*	*	*	94.7	1.1
LOS	8	42.3	3.9	44.9	3.8	44.8	3.8	46.9	3.7	44.2	3.8	76.9	2.2
Conversion	8	52.2	3.4	45.8	3.7	51.1	3.4	50.1	3.5	49.9	3.5	51.0	3.5
Incisional hernia	6	26.8	3.2	59.2	2.2	*	*	35.3	2.9	*	*	78.6	1.6
Disease Recurrence	6	52.2	2.4	41.8	2.7	*	*	52.8	2.4	*	*	53.2	2.4

SUCRA = the surface under the cumulative ranking curve; LOS = length of postoperative stay; TPLA = transperitoneal laparoscopic adrenalectomy with lateral approach; RPLA = retroperitoneal mini-invasive adrenalectomy with lateral approach; Ro-TPLA = transperitoneal robotic adrenalectomy with lateral approach; TPAA = transperitoneal laparoscopic adrenalectomy with anterior approach; SILS-LA = single-port laparoscopic adrenalectomy with lateral approach; RPA = retroperitoneal mini-invasive adrenalectomy with the posterior approach;^ = morbidity and mortality rate; * = data not available for this arm

### Primary endpoints

Regarding the primary endpoint, none of the approaches was near to an ideal approach. RPLA and RPA were the approaches having the highest probability of being the safest (69.6% and 63.0%, respectively). On average, RPLA and RPA have a mean rank near 3. TPAA, Ro-TPLA, and SILS-LA TPLA were the second choices (mean rank near 4) with SUCRA values of 45.2%, 43.4%, 43.0%, 38.5%, respectively.

### Secondary endpoints

Regarding the operative time, the RPA was the approach with the highest probability of being the fastest (SUCRA 87.5%; mean rank 1.6). The second choices were TPAA with a SUCRA value of 54.5 (mean rank 3.3). The approaches with the lowest probability of being the fastest procedures were the TPLA, RPLA, Ro-TPLA, and SILS-LA with SUCRA values of 45.1%, 45.1%, 35.7%, 32.1%, respectively.

Data about blood loss are lacking or not extractable for Ro-TPLA, TPAA, or SILS-LA. The approach having the highest probability of being those with the lowest blood loss was RPA (SUCRA 94.7%; mean rank 1.1). TPLA and RPLA were the second choices (mean rank around 3) with SUCRA values of 28.6% and 26.6%. Considering the LOS, the best approach was the RPA (SUCRA 76.9%; mean rank 2.2). TPLA, RPLA, Ro-TPLA, TPAA, and SILS-LA have equal chances in guaranteeing the shortest hospitalization (mean rank near 4, and SUCRA values around 40%). All minimally invasive approaches have similar chances of avoiding a conversion in laparotomy (SUCRA values around 50% and mean rank around 3.5). Incisional hernia and disease recurrence were not available for Ro-TPLA and SILS-LA. The approaches with the highest chances to avoid an incisional hernia were the RPA (SUCRA 78.6%; mean rank 1.6) and RPLA (SUCRA 59.2%; mean rank 2.2). The second choices were TPAA and TPLA, with SUCRA values of 35.3% and 26.8%. All approaches have similar chances of avoiding a disease recurrence (SUCRA values around 50% and mean rank around 3).

### Safety/efficacy combination

**Fig. 3:**
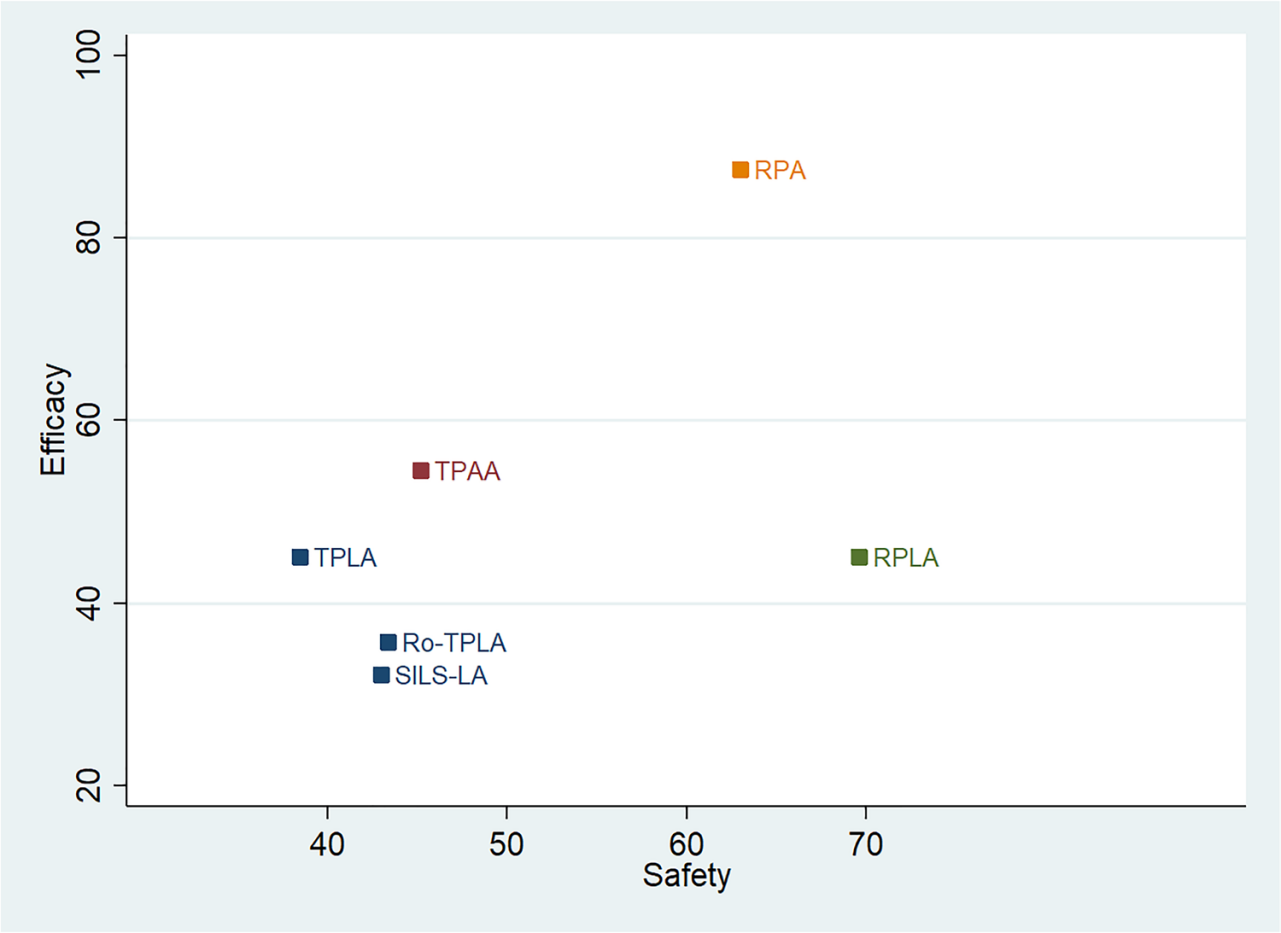
Safety/efficacy combination of all the approaches available for treating adrenal neoplasms. In the figure, the primary endpoint (safety indicator) is combined with a secondary endpoint (surrogate parameter of efficacy). Cluster rank combined the surface under the cumulative ranking curve (SUCRA) values of the composite endpoint and length of stay. On the y-axis, the SUCRA values correspond to the probability in percentages that each approach was the safest. On the x-axis, the SUCRA values correspond to the probability in percentages that each approach was most efficacious. Different colors identify the different clusters. TPLA = Transperitoneal laparoscopic lateral adrenalectomy; RPLA = retroperitoneal mini-invasive lateral adrenalectomy; Ro-TPLA = transperitoneal laparoscopic lateral adrenalectomy with robotic approach; TPAA = transperitoneal laparoscopic anterior adrenalectomy; SILS-LA = single-incision laparoscopic adrenalectomy; RPA = retroperitoneal mini-invasive posterior adrenalectomy

The combination of safety/efficacy is plotted in Fig. 3. The RPA arm was better than the other approaches, having the highest probability of being both safe and efficacious (Cophenetic correlation coefficient c = 0.95, maximum clustering gain = 461, and an optimal number of clusters = 4). The second and third choice combining high safety and efficacy were the RPLA and TPAA. The Ro-TPLA, SILS-LA, and TPLA approaches had the lowest probability of being the safest and the most efficacious.

### Inconsistency, heterogeneity, and publication bias

Inconsistency and heterogeneity for all outcomes are shown in Table 3. For all the endpoints, no source inconsistency was found within the networks. Heterogeneity was very low (τ value < 0.1) for the primary endpoint, conversion, incisional hernia, and disease recurrence rate. On the contrary, heterogeneity was high for operative time, blood loss, and LOS. The funnel plots are reported in Supplementary Fig. 5; panels A-G. The Begg and Egger test was made only for TPLA vs. RPLA because the other direct comparisons did not present sufficient observation. Egger's tests showed a significant small effect for incisional hernia and disease recurrence (*P* = 0.005 and *P* = 0.001). However, the "trim and fill" adjustment did not show significant OR changes.

**Table 3:** Inconsistency, heterogeneity, and publication bias

Outcomes of interest	Inconsistency				τ value	Publication bias^		OR (95 CI) and Adj-OR (95 CI)^	
	Global		Local			TPLA vs. RPLA		TPLA vs. RPLA^	
	Chi-square	*P* value	Loop	RoR; *P* value		Begg	Egger		
*Composite endpoint*	1.34	0.246	No	*	< 0.1	1.000	0.813	0.36 (0.08 -1.93)	0.36 (0.08 -1.93)
*Operative time*	2.43	0.119	No	*	> 1	*	*	*	*
*Blood loss*	3.41	0.065	No	*	> 1	*	*	*	*
*LOS*	1.14	0.286	No	*	0.9	*	*	*	*
*Conversion*	0.01	0.998	No	*	< 0.1	1.000	0.910	1.25 (0.19 -8.30)	1.25 (0.19 -8.30)
*Incisional hernia*	1.91	0.167	No	*	< 0.1	1.000	0.005	0.39 (0.05 -3.17)	0.39 (0.05 -3.17)
*Disease recurrence*	0.01	0.998	No	*	< 0.1	0.296	0.001	1.40 (0.17- 11.80)	1.40 (0.17- 11.80

TPLA = transperitoneal laparoscopic adrenalectomy with lateral approach; RPLA = retroperitoneal mini-invasive adrenalectomy with lateral approach; RoR = ratio of odds ratios; LOS = length of postoperative stay; * = not computable; OR = odds ratio; Adj-OR = adjusted odds ratio based on trim and fill approach; 95 CI = confidence interval at 95%; ^ = publication bias was analyzed only for TPLA vs. RPLA comparison because the other direct comparisons did not contain a sufficient number of observations

### Meta-regression analysis

Meta-regression analysis was carried out for the LOS operative time and blood loss, and is reported in Table 4 and supplementary **a, and b**. None of the covariates explained the relatively high heterogeneity (*P* > 0.05 for all covariates).

**Table 4:** Meta-regression analysis for the length of stay

Covariates	Postoperative stay SMD (95 CI; P value)						
	OA	TPLA	RPLA	Ro-TPLA	*TPAA*	*SILS-LA*	*RPA*
Country (Western vs. Eastern)	Referent	3 (± 153; 0.984)	1.6 (± 153; 0.991)	*	*	*	4.1 (± 153; 0.979)
Proportion of male patients (RR)	Referent	15.1 (± 200.2; 0.940)	12.8 (± 200.2; 0.949)	*	*	*	0.9 (± 200.5; 0.997)
Difference in age (SMD)	Referent	-11.3(± 110.9;0.919)	-22.1 (± 111;0.842)	*	*	*	-44.6 (± 115.3;0.699)
Difference in BMI (SMD)	Referent	-3.2 (± 30; 0.917)	-1.8 (± 30.6; 0.953)	*	*	*	*
Difference in tumor size (SMD)	Referent	12.1 (± 366.1;0.974)	16.8 (± 366.1;0.963)	*	*	*	2.1 (± 366.2;0.995)
Malignant tumor (No vs. Yes)	Referent	2.9 (± 31.8;0.925)	3.1 (± 31.8;0.997)	*	*	*	*
Phaeochromocytomas (No vs. Yes)	Referent	-2.8 (± 152;0.985)	-38.4 (± 3067;0.990)	*	*	*	*
Bilateral tumor (No vs. Yes)	Referent	2.4 (± 316.3;0.998)	3.8 (± 316;0.990)	*	*	*	*
Proportion of right adrenalectomy (RR)	Referent	*	*	*	*	*	*
Healthcare system (National vs. Insurance-based)	Referent	2.9 (± 31.3;0.924)	3.1 (± 31.4;0.924)	*	*	*	3.9 (± 31.4;0.899)
Study quality (low risk vs some concerns)	Referent	-3.2 (± 123.1;0.979)	*	*	*	*	*

RR = risk ratio; SMD = standard mean difference; TPLA = transperitoneal laparoscopic adrenalectomy with lateral approach; RPLA = retroperitoneal mini-invasive adrenalectomy with lateral approach; Ro-TPLA = transperitoneal robotic adrenalectomy with lateral approach; OA = open adrenalectomy; TPAA = transperitoneal laparoscopic adrenalectomy with anterior approach; SILS-LA = single-port laparoscopic adrenalectomy with lateral approach; RPA = retroperitoneal mini-invasive adrenalectomy with the posterior approach; SMD = standardized mean difference; * = not computable

## Discussion

Our study suggests that RPA and RPLA can be considered the best choices for patients who are candidate for minimally invasive adrenalectomy. These results were sustained by a meta-analysis that included only RCTs for the first time. Moreover, we analyzed the studies in a network format. Besides, even if several meta-analysis [28–46] has been produced in the last 20 years, all were affected by two major limitations: a) the bias due to non-randomized studies included; b) the difficulty to obtain clear evidence about the best technique due to the multi-arm setting. Our systematic review included only 6 phase II/III RCTs involving 359 patients because all high-risk studies were discharged.

The meta-analytic population well represents the patient candidate for minimally invasive adrenalectomy, with a mean age of 50 years and a mean size of the lesions near 3 cm. Our systematic review confirmed that the most studied minimally invasive techniques were the transperitoneal approaches (TPLA in particular), representing almost 50% of the entire cohort, followed by the retroperitoneal ones (RPA and RPLA, near 30% of the procedures). Due to their limited popularity, the robotic or single-incision approaches are rarely studied in high-quality studies (RCTs). These data confirmed those of a recent large multicentric study of Pavan et al. [47], in which the conventional laparoscopic approach resulted in the preferred way (46% of cases) compared to robotic or single-incision approaches.

Moreover, despite the popularity of TPLA, our results suggest that RPA and RPLA seem to be the minimally invasive approaches that guarantee the lowest morbidity rate. The remaining techniques have a very similar chance of being the best choice (near 40–50%). This datum is robust because, despite a certain degree of heterogeneity among the included studies (exclusion criteria and type of disease), the evaluation of the network’s coherence did not demonstrate a source of inconsistency.

These results were different from those reported by Heger et al. [35], in which the inclusion of non-randomized studies prevents seeing the differences between the transperitoneal and retroperitoneal approaches. Moreover, the large difference in morbidity observed by Heger et al. [35] in favor of the robotic approach disappeared, and this phenomenon was probably due to the selection bias of non-randomized studies. Other advantages of the retroperitoneal approach were observed for some secondary endpoints (OP, blood loss, LOS, and incisional hernia).

On the contrary, all minimally invasive techniques were similar as concern conversion rate and disease recurrence. Once again, the high quality of included studies in our meta-analysis provided us different results from Heger et al. [35]^.^ First, the OP was considered the Achilles' heel of the minimally invasive approach, but our meta-analysis did not confirm this limitation. Even if some approaches, such as robotic or SILS-based, could result slightly slower than others, these differences were minimal. Indeed, comparative studies' randomized design probably minimized the differences due to the selection bias, incomplete learning curve, or group imbalance. The only approach superior to the other seems to be the RPA. The reason for that was well underlined by Barczynski et al. [26]: the posterior technique offers direct access to the surgical site, making the operation very fast and easy. The lateral approaches (retroperitoneoscopic or transperitoneal) imply a less direct approach due to the necessity to dissect the adrenal gland from the kidney. About blood loss, our data confirmed the superiority of any minimally invasive approaches compared with the laparotomic one and established a clear hierarchy among the minimally invasive options in favor of retroperitoneal access. This could be explained considering that the adrenal glands are retroperitoneal, and the transperitoneal approach requires a large mobilization of the intra-peritoneal organs. Considering the LOS and incisional hernia, our study confirmed that all minimally invasive approaches were superior to the open, but this difference was more remarkable between the open and RPA approach. The explanation of these data could be found in the not opening of the peritoneal cavity using the retroperitoneal approach. All results seem to be robust because, despite a certain degree of heterogeneity among the included studies (exclusion criteria and type of disease), the evaluation of the network’s coherence did not demonstrate the presence of inconsistency. Significant heterogeneity was found for some secondary endpoints such as LOS, blood loss, and operative time. The meta-regression did not explain the reasons for heterogeneity. However, several non-measurable or non-extractable factors could influence these parameters, such as the modality of blood loss or operative time measurement or learning curve.

This study had some limitations. Firstly, the number of included patients was smaller than other meta-analyses available [28–46]. Nonetheless, the present study remains the only meta-analysis including exclusively RCTs, providing high-quality evidence about minimally invasive adrenalectomy.

Secondly, the learning curve differences among the included studies could influence the outcomes related to the surgeon's skills. Indeed, it is well known that OP could vary for many factors, including the surgeon's experience or patient characteristics [48]. Thus, the results about OP should be interpreted with caution even if the meta-regression analysis did not discover heterogeneity sources. Thirdly, the included studies were conducted in different countries with different healthcare systems. These differences could influence some critical outcomes, such as the LOS. Indeed, the LOS presented a certain degree of heterogeneity, even if the meta-regression analysis did not show any factor able to influence the LOS results. Finally, despite the lack of inconsistency, the eight studies included have some differences in the type of disease, inclusion, and exclusion criteria.

In conclusion**,** our study demonstrated with high-quality evidence that RPA could represent the best option for patients who are candidate to minimally invasive adrenalectomy. RPLA was a valid alternative to the posterior approach. Even when robotic platform was employed, the transperitoneal approach was a sub-optimal choice. However, these approaches should not be abandoned because a tailored approach could be required in particular settings such as paraganglioma, large adrenal masses, or hereditary syndromes.

## Supplementary Information

Below is the link to the electronic supplementary material.

Supplementary file1 (DOCX 18 KB)

Supplementary file2 (DOCX 17 KB)

Supplementary file3 (DOCX 26 KB)

Supplementary file4 (DOCX 23 KB)

Supplementary file5 (DOCX 56628 KB)

Supplementary file6 (DOCX 32 KB)
