# Supplementary material for: Minimally invasive adrenalectomy: a comprehensive systematic review and network meta-analysis of phase II/III randomized clinical controlled trials
Source: Langenbecks Arch Surg. 2022 Jan 12;407(1):285–96. doi: 10.1007/s00423-022-02431-w (PMC8847275; doi:10.1007/s00423-022-02431-w)
Supplement: Supplementary file 1 — Supplementary file1 (DOCX 18 KB) [file 423_2022_2431_MOESM1_ESM.docx]

| **First Author/year** | **Clusters** | | | | | | |
| --- | --- | --- | --- | --- | --- | --- | --- |
|  | **TPLA**  **(Arm B)** | **RPLA**  **(Arm C)** | **Ro-TPLA**  **(Arm D)** | **TPAA**  **(Arm E)** | **SILS-LA**  **(Arm F)** | **RPA**  **(Arm G)** |  |
| Fernandez-Cruz et al. 1996 ^20^ | 10 | 11 | - | - | - | - |  |
| Morino et al. 2004 ^21^ | 10 | - | 10 | - | - | - |  |
| Rubinstein et al. 2011 ^22^ | 25 | 32 | - | - | - | - |  |
| Lezoche et al. 2009 ^23^ | 25 | - | - | 25 | - | - |  |
| Vidal et al. 2012 ^24^ | 20 | - | - | - | 20 | - |  |
| Mohammadi-Fallah et al. 2013 ^25^ | 11 | 12 | - | - | - | - |  |
| Barczynski et al. 2014 ^26^ | 32 | - | - | - | - | 33 |  |
| Chai et al. 2019 ^27^ | 42 | - | - | - | - | 41 |  |
| Total | 175 | 55 | 10 | 25 | 20 | 74 |  |

**Supplementary Table 1.** Characteristics of the nine studies included

**Legend:** TPLA= transperitoneal laparoscopic adrenalectomy with lateral approach; RPLA= retroperitoneal mini-invasive adrenalectomy with lateral approach; Ro-TPLA= transperitoneal robotic adrenalectomy with lateral approach; TPAA= transperitoneal laparoscopic adrenalectomy with anterior approach; SILS-LA= Single-port laparoscopic adrenalectomy with lateral approach; RPA= Retroperitoneal mini-invasive adrenalectomy with the posterior approach.
