# Supplementary material for: Minimally invasive adrenalectomy: a comprehensive systematic review and network meta-analysis of phase II/III randomized clinical controlled trials
Source: Langenbecks Arch Surg. 2022 Jan 12;407(1):285–96. doi: 10.1007/s00423-022-02431-w (PMC8847275; doi:10.1007/s00423-022-02431-w)
Supplement: Supplementary file 2 — Supplementary file2 (DOCX 17 KB) [file 423_2022_2431_MOESM2_ESM.docx]

| First  Author | Age, year  (SMD, 95 %CI) | Male gender  (RR, 95 % CI) | Tumor size  (RR, 95 % CI) | BMI  (SMD, 95 % CI) | Right lesion  (RR, 95 % CI) |
| --- | --- | --- | --- | --- | --- |
| Fernandez-Cruz et al. 1996 ^20^ | -0.13 (-0.55 to 0.28) | 0.18 (0.01 to 3.18) | 0.20 (-0.78 to 1.18) | NE | 1.43 (0.17 to 11.76) |
| Morino et al. 2004 ^21^ | 0 (-0.42 to 0.42) | 1.00 (0.42 to 2.40) | 0.20 (-1.03 to 1.43) | -3 (-5.63 to -0.37)* | 1.00 (0.49 to 2.23) |
| Rubinstein et al. 2011 ^22^ | 0 (-0.23 to 0.51) | 0.78 (0.44 to 1.37) | -0.10 (-0.49 to 0.29) | 1 (-0.05 to 2.05) | 0.68 (0.32 to 1.42) |
| Lezoche et al. 2009 ^23^ | 0.08 (-0.32 to 0.42) | 0.81 (0.41 to 1.62) | 0.20 (-0.44 to 0.84) | -1 (-2.66 to 0.66) | NE |
| Vidal et al. 2012 ^24^ | 0.50 (-0.32 to 0.42) | 0.63 (0.25 to 1.58) | 0.10 (-0.24 to 0.44) | NE | NE |
| Mohammadi-Fallah et al. 2013 ^25^ | 0 (-0.42 to 0.42) | 0.84 (0.33 to 2.18) | -0.10 (-0.82 to 0.62) | 0 (-2.87 to 2.87) | 1.08 (0.46 to 2.51) |
| Barczynski et al. 2014 ^25^ | 0.03 (-0.37 to 0.43) | 1.16 (0.51 to 2.63) | 0.20 (0.05 to 0.35) | 0 (-0.49 to 0.49) | 0.98 (0.55 to 1.74) |
| Chai et al. 2019 ^27^ | 0 (-0.40 to 0.40) | 1.09 (0.61 to 1.96) | 0.10 (-0.48 to 0.68) | 0 (-1.29 to 1.29) | 1.02 (0.59 to 1.75) |

**Supplementary Table 2.** Covariates potentially source of bias and heterogeneity in included studies

**Abbreviations:**  SMD= Standardized Mean Difference; BMI= Body Mass Index;95 % CI= confidence interval at 95 %; RR= risk ratio; NE= data not reported or not extractable; *= the difference between experimental and referent arm was significant.
