# Supplementary material for: Minimally invasive adrenalectomy: a comprehensive systematic review and network meta-analysis of phase II/III randomized clinical controlled trials
Source: Langenbecks Arch Surg. 2022 Jan 12;407(1):285–96. doi: 10.1007/s00423-022-02431-w (PMC8847275; doi:10.1007/s00423-022-02431-w)
Supplement: Supplementary file 3 — Supplementary file3 (DOCX 26 KB) [file 423_2022_2431_MOESM3_ESM.docx]

| **Rank** | **TPLA (%)** | **RPLA (%)** | **Ro-TPLA (%)** | **TPAA (%)** | **SILS-LA (%)** | **RPA (%)** |
| --- | --- | --- | --- | --- | --- | --- |
| **Morbidity** |  |  |  |  |  |  |
| *Best* | *0.4* | *26.6* | *20.5* | *18.8* | *20.9* | *12.8* |
| *2^nd^* | *2.4* | *30.3* | *13.6* | *14.0* | *12.9* | *26.8* |
| *3^td^* | *17.6* | *21.4* | *9.6* | *10.4* | *10.1* | *30.9* |
| *4^th^* | *36.3* | *12.7* | *10.3* | *11.2* | *11.3* | *20.3* |
| *5^th^* | *35.0* | *7.2* | *16.3* | *17.7* | *16.4* | *7.4* |
| *Worst* | *8.3* | *1.8* | *29.7* | *30.0* | *28.4* | *1.8* |
|  |  |  |  |  |  |  |
| **Operative time** |  |  |  |  |  |  |
| *Best* | *0.3* | *6.3* | *8.6* | *17.2* | *8.2* | *59.4* |
| *2^nd^* | *8.6* | *17.6* | *14.0* | *21.6* | *13.1* | *25.1* |
| *3^td^* | *31.4* | *21.7* | *9.5* | *17.8* | *11.0* | *8.6* |
| *4^th^* | *36.9* | *20.5* | *13.7* | *13.6* | *12.3* | *3.0* |
| *5^th^* | *19.0* | *21.4* | *21.9* | *14.6* | *20.7* | *2.4* |
| *Worst* | *3.8* | *12.5* | *32.3* | *15.2* | *34.7* | *1.5* |
|  |  |  |  |  |  |  |
| ***Blood Loss*** |  |  |  |  |  |  |
| *Best* | 1.8 | 7.1 | * | * | * | 91.1 |
| *2^nd^* | 51.9 | 41.1 | * | * | * | 7.0 |
| *Worst* | 46.3 | 51.8 | * | * | * | 1.9 |
|  |  |  |  |  |  |  |
| **LOS** |  |  |  |  |  |  |
| *Best* | 0.5 | 7.5 | 15.8 | 17.6 | 16.0 | 42.5 |
| *2^nd^* | 7.8 | 15.2 | 15.8 | 14.7 | 17.4 | 29.1 |
| *3^td^* | 26.0 | 20.9 | 12.6 | 12.8 | 13.9 | 13.8 |
| *4^th^* | 37.1 | 19.2 | 11.9 | 12.1 | 11.9 | 7.8 |
| *5^th^* | 22.5 | 23.4 | 15.3 | 19.7 | 14.6 | 4.5 |
| *Worst* | 6.1 | 13.7 | 28.6 | 23.1 | 26.2 | 2.3 |
|  |  |  |  |  |  |  |
| ***Conversion*** |  |  |  |  |  |  |
| *Best* | 2.0 | 7.1 | 23.8 | 25.0 | 22.8 | 19.3 |
| *2^nd^* | 19.1 | 15.7 | 15.0 | 17.2 | 15.8 | 17.2 |
| *3^td^* | 31.6 | 19.8 | 12.3 | 11.6 | 11.3 | 13.4 |
| *4^th^* | 29.4 | 20.9 | 12.5 | 8.8 | 11.0 | 17.4 |
| *5^th^* | 15.4 | 23.4 | 16.4 | 13.7 | 14.1 | 17.0 |
| *Worst* | 2.5 | 13.1 | 20.0 | 23.7 | 25.0 | 15.7 |
|  |  |  |  |  |  |  |
| **Incisional Hernia** |  |  |  |  |  |  |
| *Best* | 0.8 | 23.8 | * | 17.4 | * | 58.0 |
| *2^nd^* | 13.8 | 42.2 | * | 16.8 | * | 27.2 |
| *3^td^* | 49.9 | 23.8 | * | 15.5 | * | 10.8 |
| *Worst* | 35.5 | 10.2 | * | 50.3 | * | 4.0 |
|  |  |  |  |  |  |  |
| **Disease recurrence** |  |  |  |  |  |  |
| *Best* | 17.2 | 16.0 | * | 35.8 | * | 31.0 |
| *2^nd^* | 39.5 | 23.1 | * | 14.6 | * | 22.8 |
| *3^td^* | 35.4 | 28.7 | * | 15.2 | * | 20.7 |
| *Worst* | 7.9 | 32.2 | * | 34.4 | * | 25.5 |
|  |  |  |  |  |  |  |

**Supplementary Table 3-** The ranking of the approaches for all outcomes.

**Legend:** The probability in percentages of the approaches ranking from best to worst is reported in the column. LOS= length of postoperative stay; TPLA= transperitoneal laparoscopic adrenalectomy with lateral approach; RPLA= retroperitoneal mini-invasive adrenalectomy with lateral approach; Ro-TPLA= transperitoneal robotic adrenalectomy with lateral approach; OA= Open adrenalectomy; TPAA= transperitoneal laparoscopic adrenalectomy with anterior approach; SILS-LA= Single-port laparoscopic adrenalectomy with lateral approach; RPA= Retroperitoneal mini-invasive adrenalectomy with the posterior approach; *= data not available for this arm.
