# Supplementary material for: Minimally invasive adrenalectomy: a comprehensive systematic review and network meta-analysis of phase II/III randomized clinical controlled trials
Source: Langenbecks Arch Surg. 2022 Jan 12;407(1):285–96. doi: 10.1007/s00423-022-02431-w (PMC8847275; doi:10.1007/s00423-022-02431-w)
Supplement: Supplementary file 4 — Supplementary file4 (DOCX 23 KB) [file 423_2022_2431_MOESM4_ESM.docx]

| Covariates | Operative time  SMD (95 CI; P-value) | | | | | | |
| --- | --- | --- | --- | --- | --- | --- | --- |
|  | **OA** | **TPLA** | **RPLA** | **Ro-TPLA** | ***TPAA*** | ***SILS-LA*** | ***RPA*** |
| Country (Western vs. Eastern) | Referent | 22.8 (±4595; 0.996) | 8.7 (±4595; 0.998) | * | * | * | 56.8 (±4595; 0.990) |
| Proportion of male patients (RR) | Referent | * | * | * | * | * | * |
| Difference in age (SMD) | Referent | -124.63 (±13007;0.992) | -305.63 (±13008;0.981) | * | * | * | -125.97 (±13009;0.923) |
| Difference in BMI (SMD) | Referent | 27.1 (±902; 0.976) | -2.8 (±902; 0.997) | * | * | * | * |
| Difference in tumor size (SMD) | Referent | * | * | * | * | * | * |
| Malignant tumor (No vs. Yes) | Referent | 26.9 (±3731;0.994) | 12.7 (±3731;0.997) | * | * | * | * |
| Phaeochromocytomas (No vs. Yes) | Referent | -14.3 (±3067;0.996) | -38.4 (±3067;0.990) | * | * | * | * |
| Bilateral tumor (No vs. Yes) | Referent | 13.6 (±6448;0.998) | 37.6 (±6448;0.995) | * | * | * | * |
| Proportion of right adrenalectomy (RR) | Referent | * | * | * | * | * | * |
| Health care system (National vs. Insurance-based) | Referent | 27.6 (±20619;0.999) | 13.3 (±20619;0.999) | * | * | * | * |
| Study quality (low risk vs some concerns) | Referent | -27.1 (±941; 0.977) | * | * | * | * | * |

**Supplementary Table 4a- Meta-regression analysis for operative time**

**Legend:** RR= Risk Ratio; SMD=standard mean difference; TPLA= transperitoneal laparoscopic adrenalectomy with lateral approach; RPLA= retroperitoneal mini-invasive adrenalectomy with lateral approach; Ro-TPLA= transperitoneal robotic adrenalectomy with lateral approach; OA= Open adrenalectomy; TPAA= transperitoneal laparoscopic adrenalectomy with anterior approach; SILS-LA= Single-port laparoscopic adrenalectomy with lateral approach; RPA= Retroperitoneal mini-invasive adrenalectomy with the posterior approach; SMD= standardized mean difference; RR= Risk ratio; *= not computable

| Covariates | Blood loss  SMD (95 CI; P-value) | | | |
| --- | --- | --- | --- | --- |
|  | **OA** | **TPLA** | **RPLA** | ***RPA*** |
| Country (Western vs. Eastern) | Referent | 117.5 (±6794; 0.984) | 117.1 (±5794; 0.984) | 56.8 (±4595; 0.990) |
| Proportion of male patients (RR) | Referent | 301.1 (±7655;0.969) | 270.3 (±7655;0.972) | -270.4 (±7655;0.972) |
| Difference in age (SMD) | Referent | -510.7 (±9952;0.959) | -664.6 (±9955;0.947) | -1844.1 (±9953;0.853) |
| Difference in BMI (SMD) | Referent | -11.7 (±6610; 0.999) | -11.7 (±6610; 0.999) | * |
| Difference in tumor size (SMD) | Referent | 470.7 (±10166;0.963) | 537.3 (±10167;0.958) | 70.7 (±10166;0.994) |
| Malignant tumor (No vs. Yes) | Referent | 115.9 (±6008;0.985) | 110.2 (±6008;0.985) | * |
| Phaeochromocytomas (No vs. Yes) | Referent | -105.7 (±27783;0.997) | -125.8 (±27783;0.996) | * |
| Bilateral tumor (No vs. Yes) | Referent | 105.5 (±25105;0.997) | 125.5 (±25105;0.996) | * |
| Proportion of right adrenalectomy (RR) | Referent | -480.7 (±4397;0.913) | -472.1(±4397;0.915) | 519.3(±4395;0.906) |
| Health care system (National vs Insurance-based) | Referent | 116.9 (±4772;0.980) | 114.8 (±4772;0.981) | 156.9 (±4772;0.974) |
| Study quality (low risk vs some concerns) | Referent | -129.1 (±2912;0.965) | * | * |

**Supplementary Table 4b- Meta-regression analysis for blood loss**

**Legend:** RR= Risk Ratio; SMD=standard mean difference; TPLA= transperitoneal laparoscopic adrenalectomy with lateral approach; RPLA= retroperitoneal mini-invasive adrenalectomy with lateral approach; Ro-TPLA= transperitoneal robotic adrenalectomy with lateral approach; OA= Open adrenalectomy; RPA= Retroperitoneal mini-invasive adrenalectomy with the posterior approach; *= not computable
