# Supplementary material for: Minimally invasive adrenalectomy: a comprehensive systematic review and network meta-analysis of phase II/III randomized clinical controlled trials
Source: Langenbecks Arch Surg. 2022 Jan 12;407(1):285–96. doi: 10.1007/s00423-022-02431-w (PMC8847275; doi:10.1007/s00423-022-02431-w)
Supplement: Supplementary file 5 — Supplementary file5 (DOCX 56628 KB) [file 423_2022_2431_MOESM5_ESM.docx]

**Supplementary methods**

*Information sources, search and eligibility criteria*

A systematic literature search was conducted using Medline, The Cochrane Library, Scopus and the ISI-Web of Science databases to identify all randomized studies published about mini-invasive adrenalectomy. The search strategy used was the following: (“Laparoscopic adrenalectomy OR retroperitoneoscopic adrenalectomy OR robotic adrenalectomy OR single-incision adrenalectomy AND randomized controlled trial”). The related articles found were used to broaden the search, and all the abstracts, studies and citations obtained were reviewed. In addition, the references of all the studies included were screened for any other potentially relevant studies. The last search was conducted on 17 January 2021. Thomson Reuters Endnote version X7 was used to manage the literature results

*Study selection, inclusion and exclusion criteria*

For inclusion in the meta-analysis, the studies analyzed had to meet the following criteria: 1) comparative study between two minimal invasive approach or between one minimal invasive versus open technique for adrenalectomy; 2) randomized design; 3) reporting data and details concerning at least the mortality and morbidity rate. Finally, if two studies were reported by the same institution (and/or authors) or presented risk of overlapping populations, either the most recent study or the one of higher quality was included. The following criteria were used to exclude studies: 1) nonrandomized comparative studies, guidelines, letters to the editor and abstracts without a full text; 2) randomized studies without adequate data were excluded

*Data collection process and item*

Two independent reviewers (L.A. and C.I.) carried out the data extraction using standardized data forms. All data from each eligible study was entered into a dedicated spreadsheet (Excel 2007, Microsoft Corporation®). The following data were extracted in order to describe the characteristics of each study: authors, year of publication, affiliation and country, study design, total patients randomized, male/female ratio, mean age, BMI, tumor size, malignant tumors, phaeochromocytomas rate, bilateral procedures, side of lesion, health care system. The outcomes evaluated were morbidity and mortality rate (primary endpoint), operative time, blood loss, postoperative hospital stay, conversion to the open surgery, incisional hernia, and recurrence of the disease.

*Geometry of network*

The geometry of the network was plotted using one node for each intervention arm and an edge that connected two nodes for each trial. The size of the node represents the number of patients included in each arm. The network geometry was preliminarily explored for all outcomes of interest to evaluate the presence of common nodes. When a common node was absent, the network was defined disconnected, and this condition precludes the analysis in network modality. The network was also reported in a matrix form to obtain information about the contribution of included studies.

*Risk of bias within individual studies*

The risk of bias within the individual studies was evaluated using a revised tool to assess randomized trials' bias (RoB2, latest version on July 9, 2019).^1^ Two review authors (C.R. and R.C.) independently assessed the risk of bias for each study using the criteria outlined in the Cochrane Handbook for Systematic Reviews of Interventions^2^. Any disagreements were solved by a discussion with the last authors (M.F.)

*Summary of measures*

All indirect and mixed estimates were reported as odds ratios (ORs) or mean differences (MDs) for dichotomous outcomes and continuous variables, respectively. The ORs and MDs were expressed with 95% confidence intervals (CIs). An OR with CIs crossing 1 or an MD with CIs crossing 0 indicated that the two competitive approaches were equivalent. The network estimates (indirect and mixed) were reported in the forest plot ^3^ with CIs and predicting interval (PrI). The network results were reported first as “relative ranking probability,” which represented the probability that each approach would be the best, the second, the third, and the worst with a certain degree of uncertainty for each outcome of interest. Thus, starting these values, the surface under the cumulative ranking (SUCRA) curves and mean ranks were obtained. The SUCRA value, expressed as a percentage, showed the probability, without uncertainty, that each approach would be the best option, based on the analyzed outcome represented.^4^

*Planned method of analysis, inconsistency, risk of bias across the study and additional analyses*

The PRISMA extension statement incorporating Network Meta-Analyses of Health Care Interventions was used to plan the analysis. Frequentist network meta-analysis was employed to compare all interventions for distal pancreatectomy in patients building a network for each outcome of interest.^5^ The analysis was performed in two steps: first, all pair-wise (“head-to-head”) comparisons in each network were calculated to obtain the indirect and mixed estimates. Second, we calculated relative ranking probabilities, and thus SUCRA values were obtained.^6^ The robustness of the networks was assessed by evaluating the presence of inconsistency, heterogeneity, and publication bias. The presence of inconsistency was evaluated using the “loop” approach.^7^ On the other hand, the restricted maximum likelihood method was used to estimate heterogeneity. The extent of heterogeneity in each network was evaluated by comparing the magnitude of a common heterogeneity variance for the network (tau [τ]) with an empirical distribution of heterogeneity variances, considering the range of expected treatment estimates (ORs and MDs). A τ value less than 0.1 indicated a very low level of heterogeneity while a τ value from 0.1 to 0.5 indicated a moderate level; a τ value> 0.5 to was considered a high level of heterogeneity.^8^ When the τ value was > 0.5, a multivariate meta-regression analysis was carried out to identify the reason for the heterogeneity in the outcome under study. Thus, the effects of all the covariates were reported using a beta (β) coefficient and a P-value. The algorithm adopted was based on the use of maximum residual likelihood (REML). For each covariate, we described, only when significant, the following parameters: β coefficient with standard error (SE) and R^2^. The β coefficient ± SE was related to the change of covariate value. If β was > 0 or <0, an increased o reduction of the covariate produced a positive or negative modification of OR or MD. On the other hand, the R^2^ indicated the percentage of between-study variance explained by the covariate. A two-tailed P value <0.05 was considered statistically significant. Considering the low number of included studies in previous meta-analyses, the P-value was also recalculated using Monte Carlo permutation.^9^ The number of permutations was 500 to obtain sufficient precision.^10^ Publication/reporting bias was reported using an adjusted funnel plot. Each funnel plot was tested using Begg’s test to identify whether the asymmetry was attributable to the small sample size effect. A two-sided P value <0.05 indicated a significant small sample size effect. ^11^

**Supplementary Figures**

**Supplementary Figure 1- Panel A, mortality and morbidity**

**
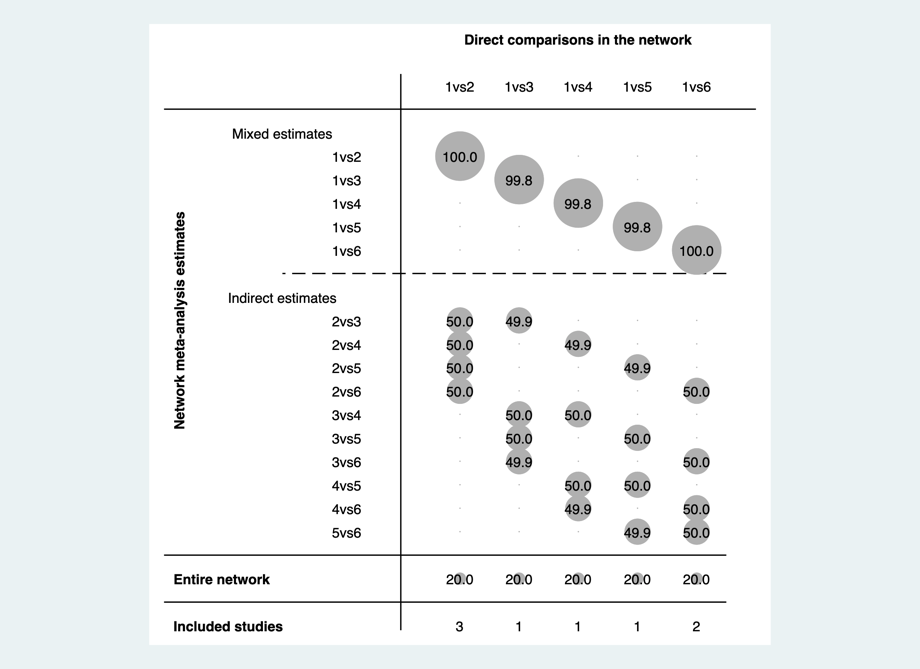
**

Legend:1= Transperitoneal lateral laparoscopic approach; 2=Retroperitoneal lateral approach; 3=Robotic transperitoneal lateral approach; 4=Transperitoneal anterior laparoscopic approach; 5= Single-incision laparoscopic approach; 6= Retroperitoneal posterior approach.

**Supplementary Figure 1- Panel B, operative time**

**
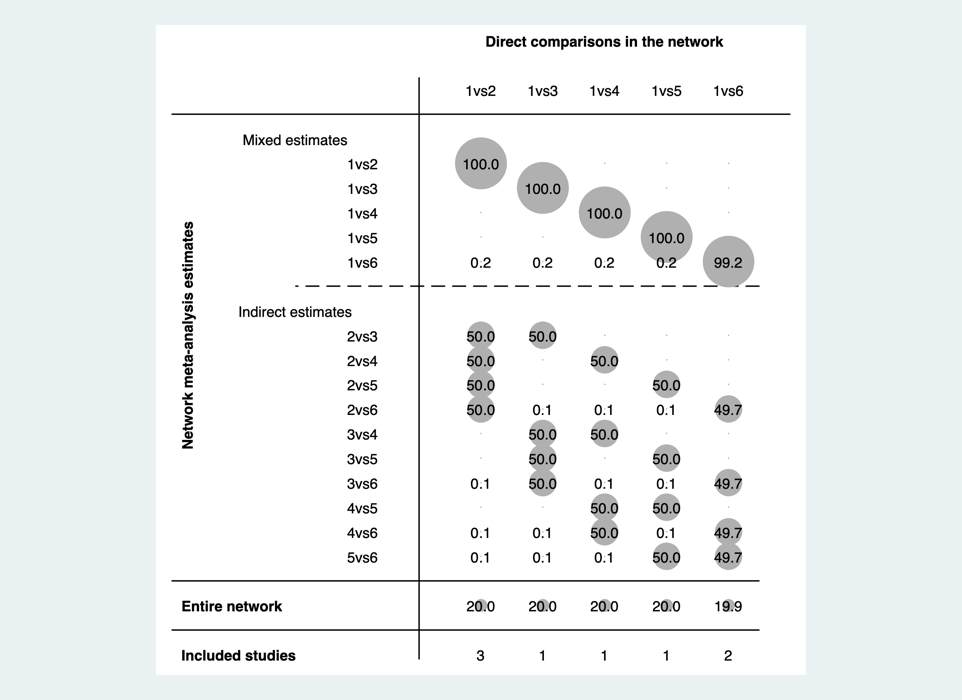
**

Legend: 1= Transperitoneal lateral laparoscopic approach; 2=Retroperitoneal lateral approach; 3=Robotic transperitoneal lateral approach; 4=Transperitoneal anterior laparoscopic approach; 5= Single-incision laparoscopic approach; 6= Retroperitoneal posterior approach.

**Supplementary Figure 1- Panel C, blood loss**

**
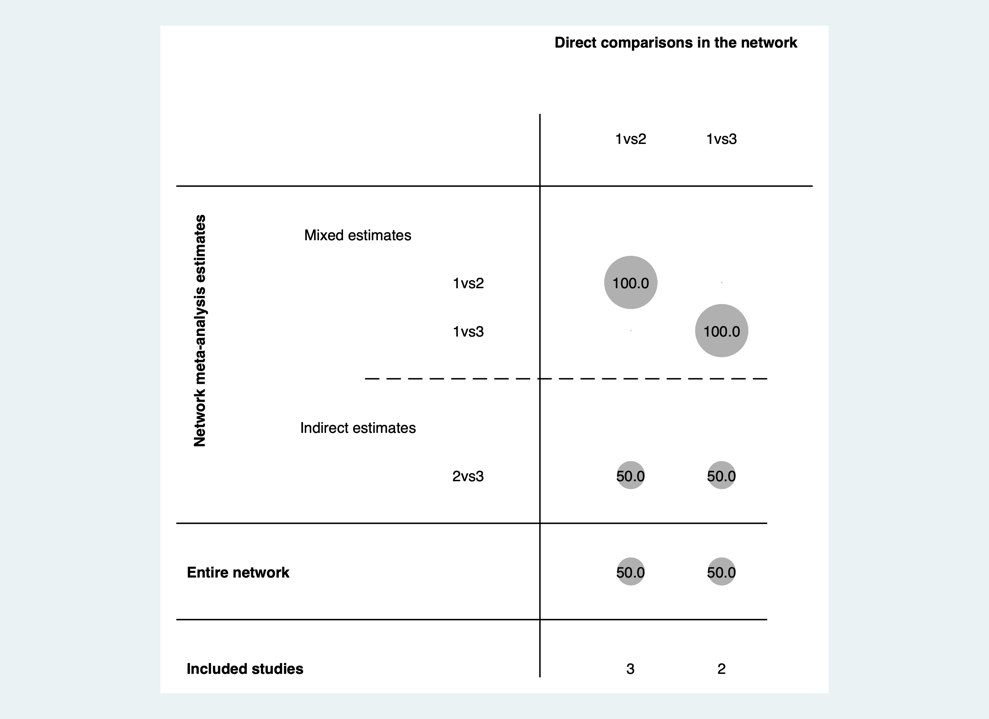
**

Legend: 1=Transperitoneal lateral laparoscopic approach; 2=Retroperitoneal lateral approach; 3= Retroperitoneal posterior approach.

**Supplementary Figure 1- Panel D, length of stay**


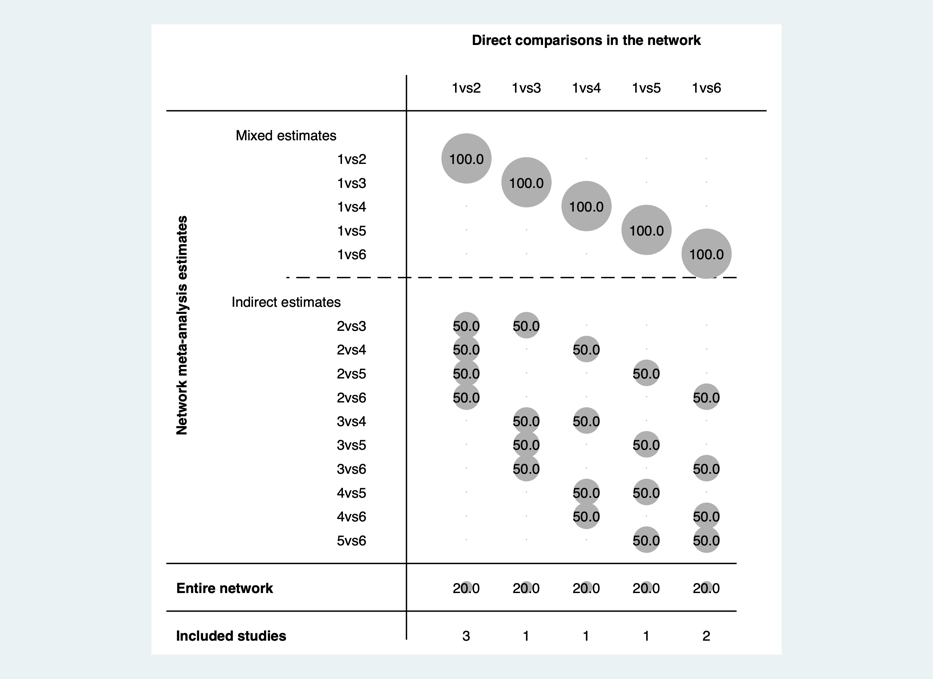


Legend: 1= Transperitoneal lateral laparoscopic approach; 2=Retroperitoneal lateral approach; 3=Robotic transperitoneal lateral approach; 4=Transperitoneal anterior laparoscopic approach; 5= Single-incision laparoscopic approach; 6= Retroperitoneal posterior approach.

**Supplementary Figure 1- Panel E, conversion**

**
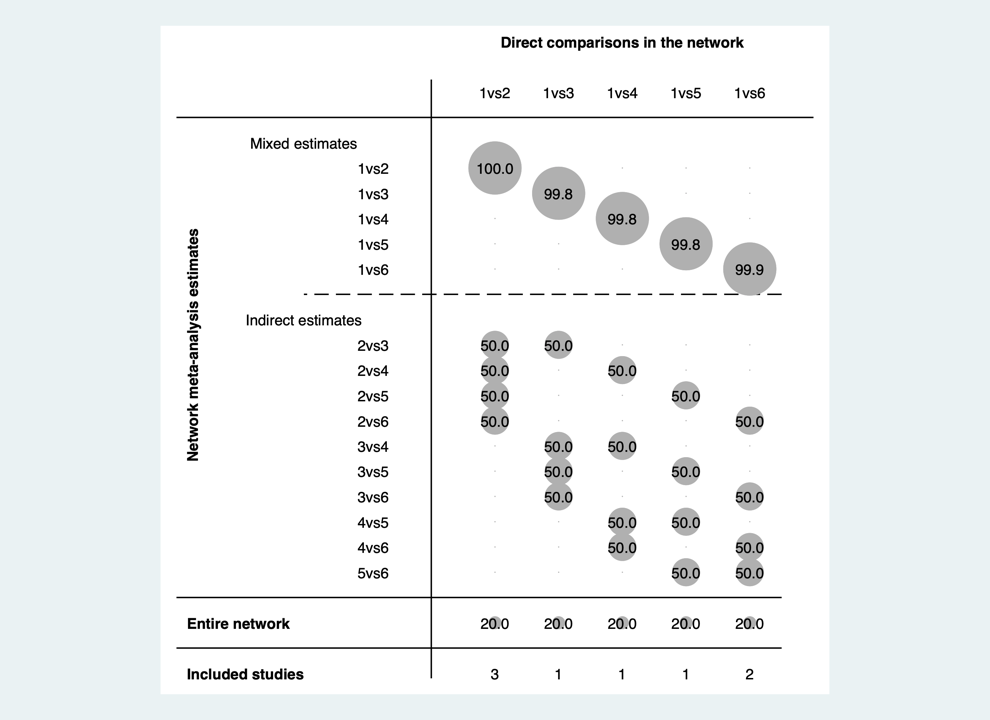
**

Legend: 1= Transperitoneal lateral laparoscopic approach; 2=Retroperitoneal lateral approach; 3=Robotic transperitoneal lateral approach; 4=Transperitoneal anterior laparoscopic approach; 5= Single-incision laparoscopic approach; 6= Retroperitoneal posterior approach.

**Supplementary Figure 1- Panel F, incisional hernia**

**
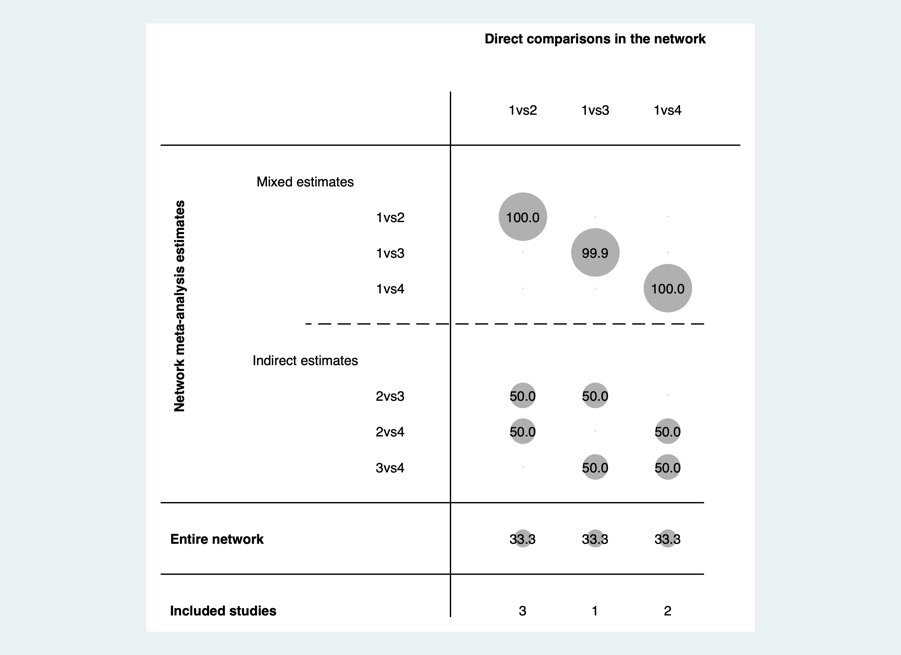
**

Legend: 1= Transperitoneal lateral laparoscopic approach; 2=Retroperitoneal lateral approach; 3= Transperitoneal anterior laparoscopic approach; 4= Retroperitoneal posterior approach.

**Supplementary Figure 1- Panel G, disease recurrence**

**
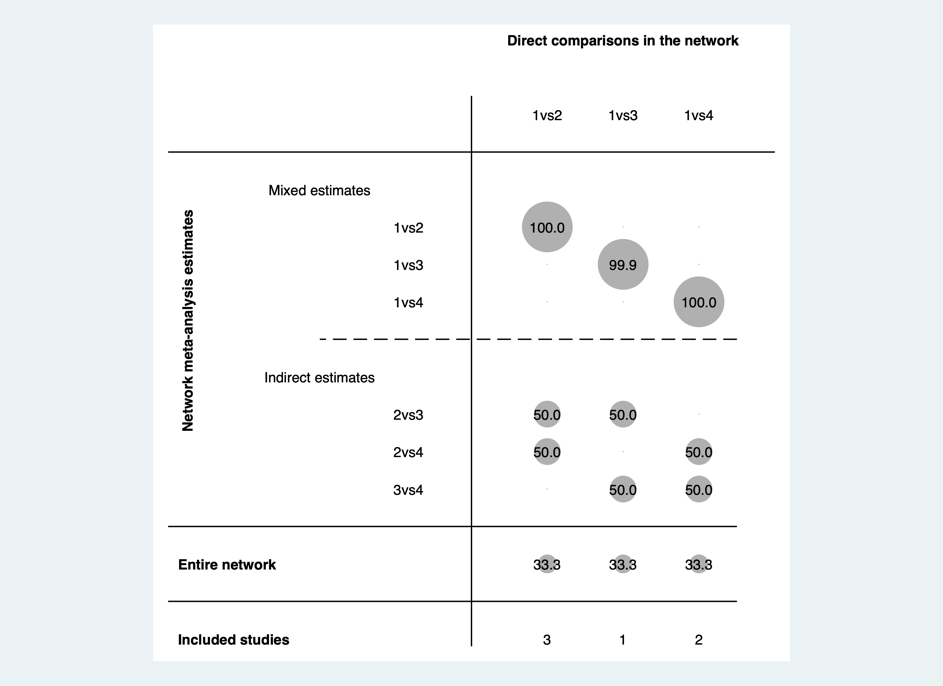
**

Legend: 1= Transperitoneal lateral laparoscopic approach; 2=Retroperitoneal lateral approach; 3= Transperitoneal anterior laparoscopic approach; 4= Retroperitoneal posterior approach.

**Supplementary Figure 2- Panel A, blood loss and incisional hernia**

**
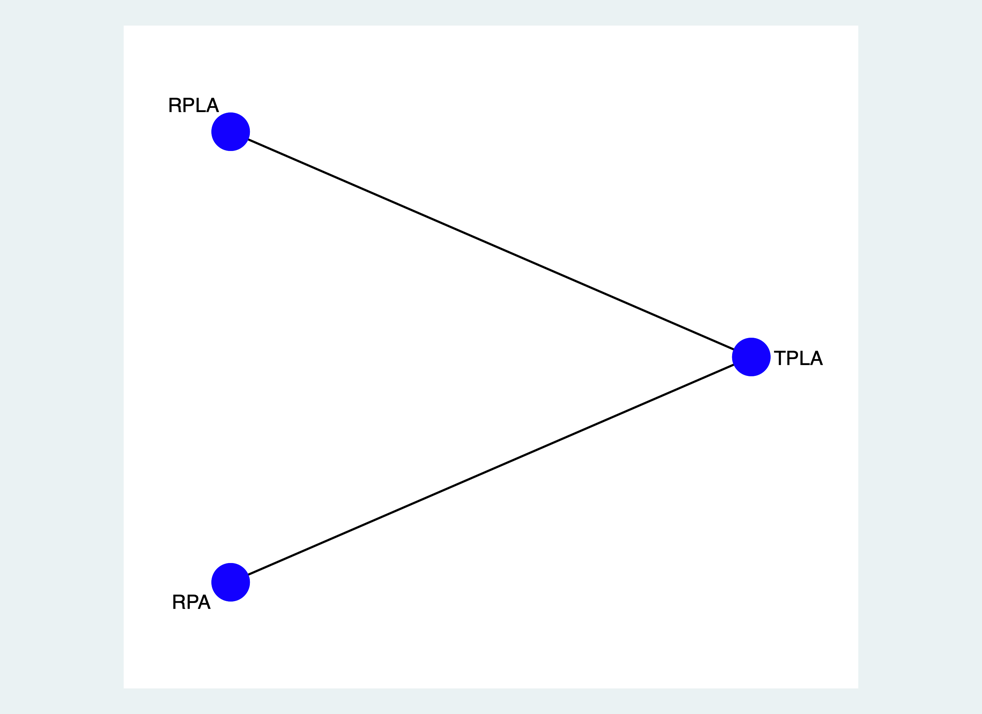
**

**Legend:** TPLA= transperitoneal lateral laparoscopic approach; RPLA= Retroperitoneoscopic lateral approach; RPA= retroperitoneal approach

**Supplementary Figure 2- Panel B, recurrence of the disease**

**
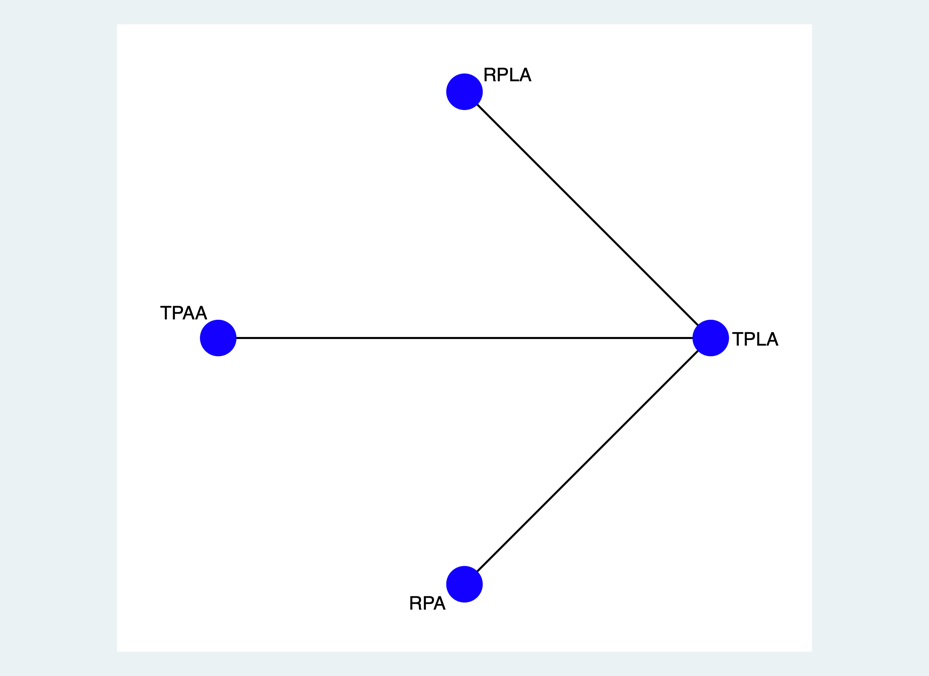
**

**Legend:** TPLA= transperitoneal lateral laparoscopic approach; RPLA= Retroperitoneoscopic lateral approach; TPAA= transperitoneal anterior laparoscopic approach; RPA= retroperitoneal approach

**Supplementary Figure 3- Panel A**

**
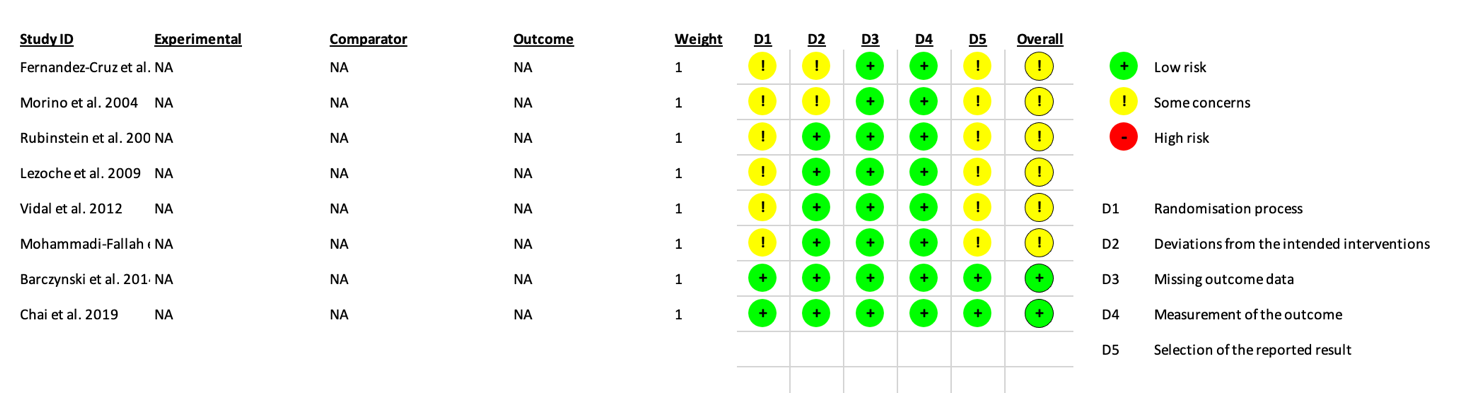
**

**Supplementary Figure 4- Panel A, morbidity and mortality**

**
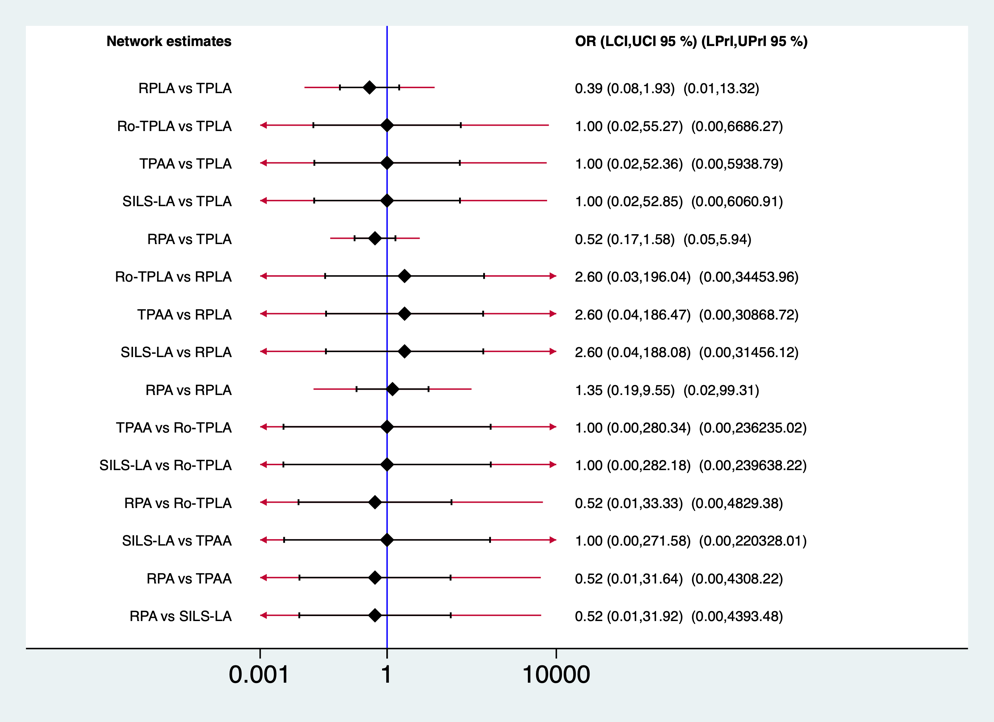
**

Legend: TPLA=Transperitoneal laparoscopic lateral adrenalectomy; RPLA= Retroperitoneal mini-invasive lateral adrenalectomy; Ro-TPLA=Transperitoneal laparoscopic lateral adrenalectomy with robotic approach; TPAA=Transperitoneal laparoscopic anterior adrenalectomy; SILS-LA= Single-incision laparoscopic adrenalectomy; RPA= Retroperitoneal mini-invasive posterior adrenalectomy; OR= odds ratio; LCI= Lower Confidence Interval; UCI=Upper Confidence Interval; LPrI= Lower predictive Interval; UPrI=Upper predictive Interval.

**Supplementary Figure 4- Panel B, operative time**

**
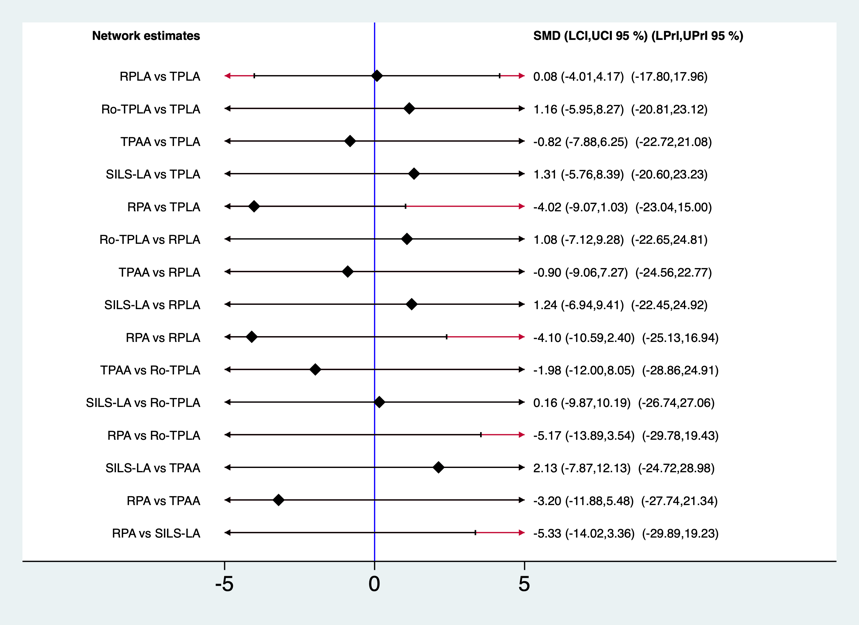
**

Legend: TPLA=Transperitoneal laparoscopic lateral adrenalectomy; RPLA= Retroperitoneal mini-invasive lateral adrenalectomy; Ro-TPLA=Transperitoneal laparoscopic lateral adrenalectomy with robotic approach; TPAA=Transperitoneal laparoscopic anterior adrenalectomy; SILS-LA= Single-incision laparoscopic adrenalectomy; RPA= Retroperitoneal mini-invasive posterior adrenalectomy; SMD= standardized mean difference; LCI= Lower Confidence Interval; UCI=Upper Confidence Interval; LPrI= Lower predictive Interval; UPrI=Upper predictive Interval.

**Supplementary Figure 4- Panel C, blood loss**

**
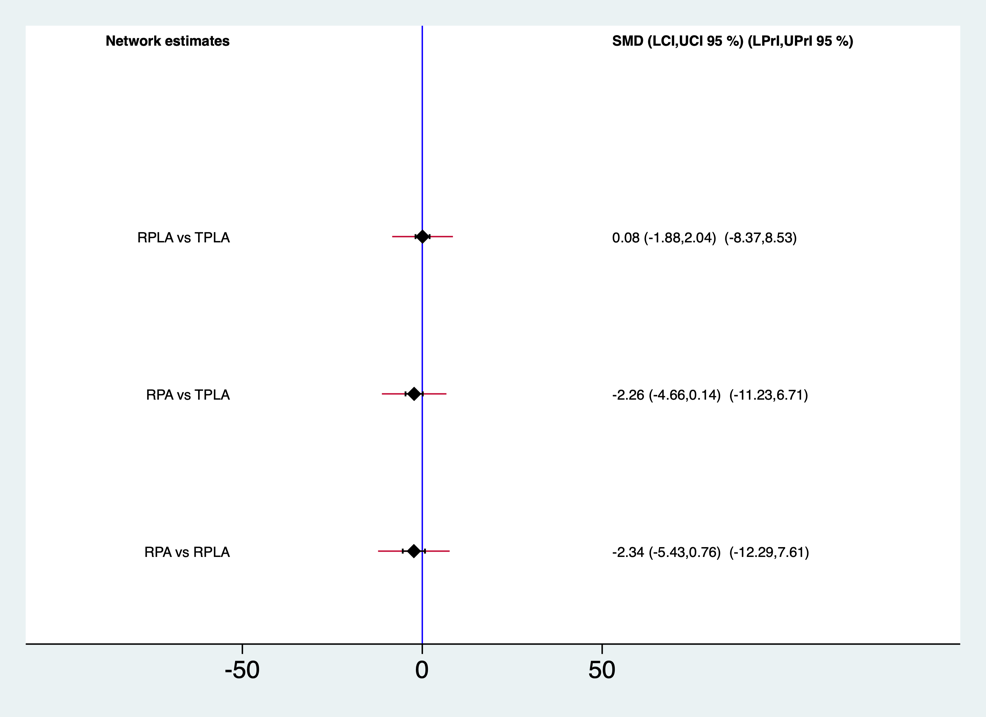
**

Legend: TPLA=Transperitoneal laparoscopic lateral adrenalectomy; RPLA= Retroperitoneal mini-invasive lateral adrenalectomy; RPA= Retroperitoneal mini-invasive posterior adrenalectomy; SMD= standardized mean difference; LCI= Lower Confidence Interval; UCI=Upper Confidence Interval; LPrI= Lower predictive Interval; UPrI=Upper predictive Interval.

**Supplementary Figure 4- Panel D, length of stay**


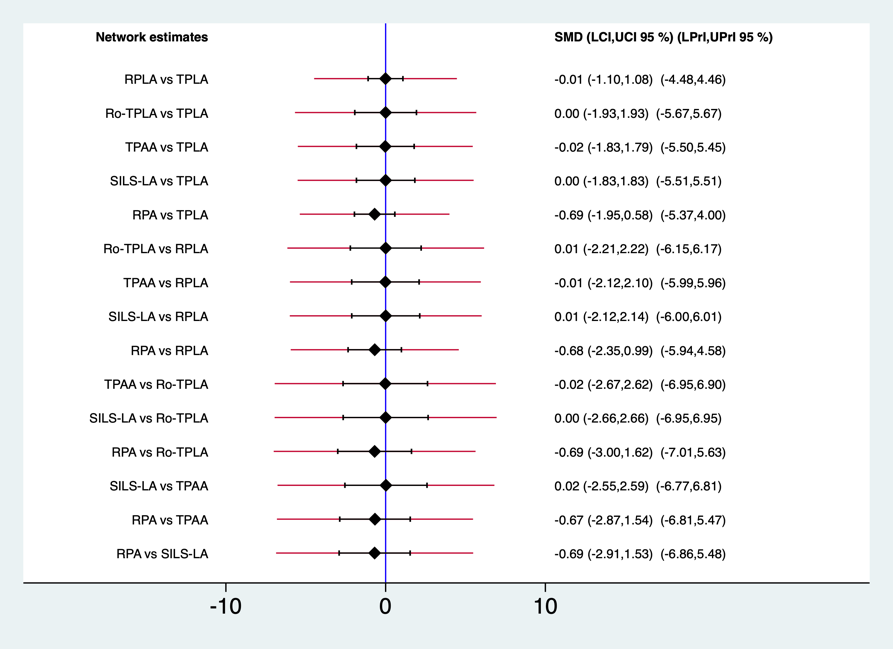


Legend: TPLA=Transperitoneal laparoscopic lateral adrenalectomy; RPLA= Retroperitoneal mini-invasive lateral adrenalectomy; Ro-TPLA=Transperitoneal laparoscopic lateral adrenalectomy with robotic approach; TPAA=Transperitoneal laparoscopic anterior adrenalectomy; SILS-LA= Single-incision laparoscopic adrenalectomy; RPA= Retroperitoneal mini-invasive posterior adrenalectomy; SMD= standardized mean difference; LCI= Lower Confidence Interval; UCI=Upper Confidence Interval; LPrI= Lower predictive Interval; UPrI=Upper predictive Interval.

**Supplementary Figure 4- Panel E, Conversion**

**
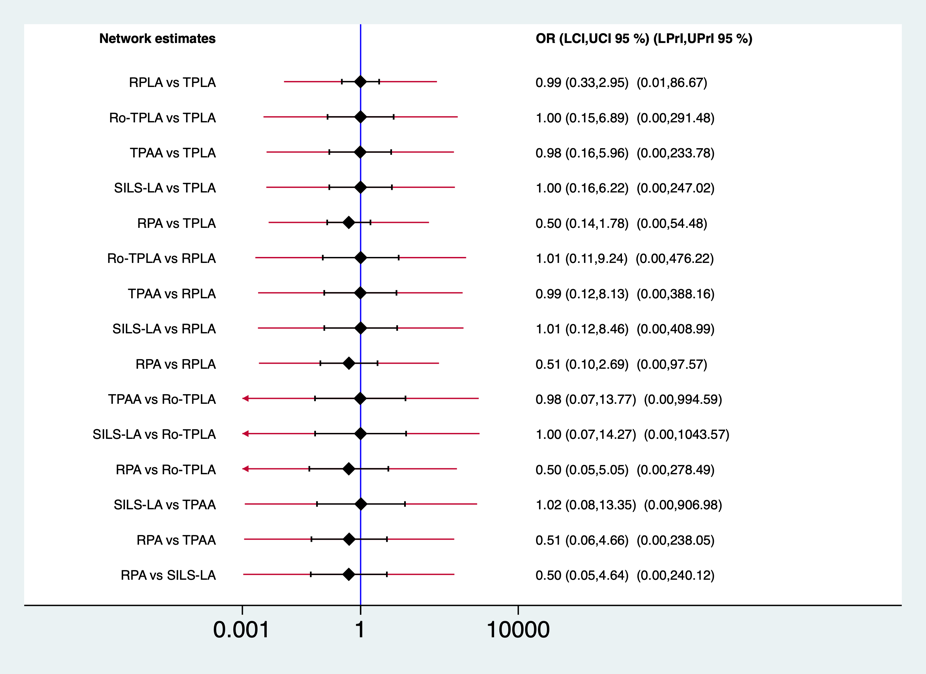
**

Legend: TPLA=Transperitoneal laparoscopic lateral adrenalectomy; RPLA= Retroperitoneal mini-invasive lateral adrenalectomy; Ro-TPLA=Transperitoneal laparoscopic lateral adrenalectomy with robotic approach; TPAA=Transperitoneal laparoscopic anterior adrenalectomy; SILS-LA= Single-incision laparoscopic adrenalectomy; RPA= Retroperitoneal mini-invasive posterior adrenalectomy;; OR= odds ratio; LCI= Lower Confidence Interval; UCI=Upper Confidence Interval; LPrI= Lower predictive Interval; UPrI=Upper predictive Interval.

**Supplementary Figure 4- Panel F, Incisional Hernia**

**
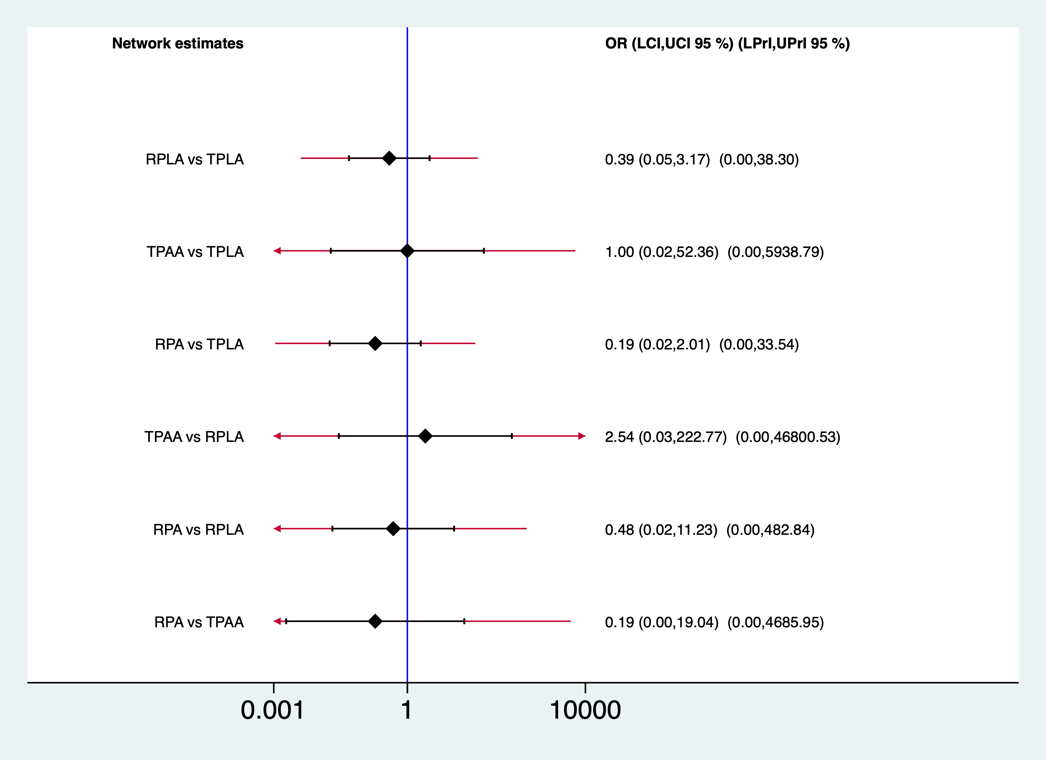
**

Legend: TPLA=Transperitoneal laparoscopic lateral adrenalectomy; RPLA= Retroperitoneal mini-invasive lateral adrenalectomy; TPAA=Transperitoneal laparoscopic anterior adrenalectomy; RPA= Retroperitoneal mini-invasive posterior adrenalectomy; OR= odds ratio; LCI= Lower Confidence Interval; UCI=Upper Confidence Interval; LPrI= Lower predictive Interval; UPrI=Upper predictive Interval.

**Supplementary Figure 4- Panel G, Disease recurrence**

**
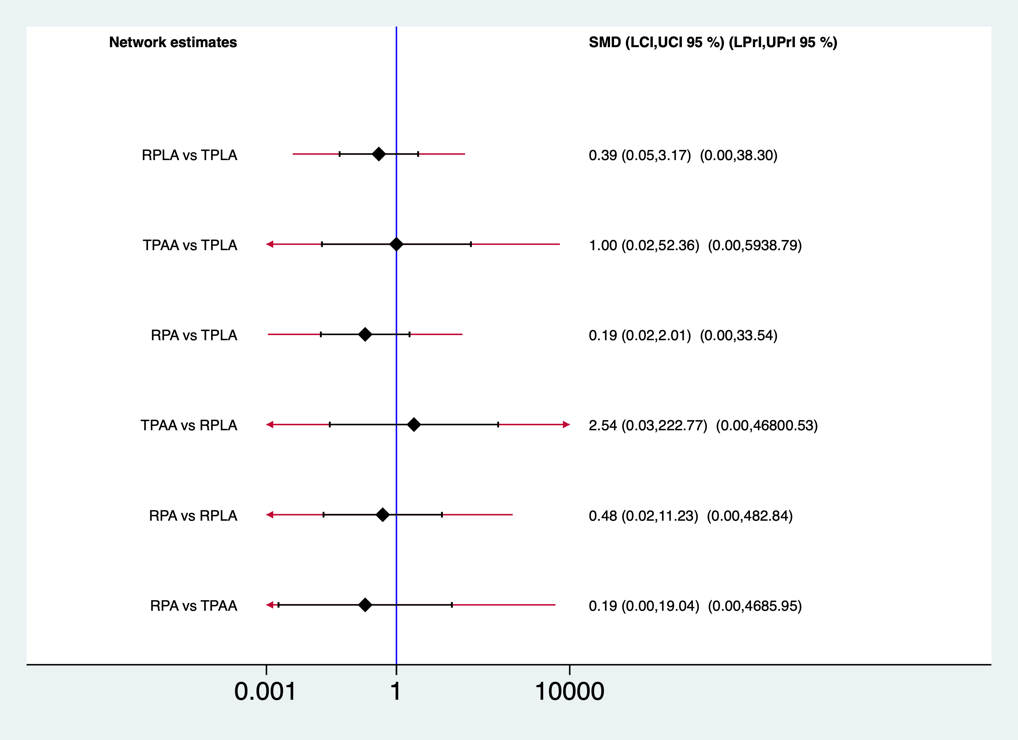
**

Legend: TPLA=Transperitoneal laparoscopic lateral adrenalectomy; RPLA= Retroperitoneal mini-invasive lateral adrenalectomy; TPAA=Transperitoneal laparoscopic anterior adrenalectomy; RPA= Retroperitoneal mini-invasive posterior adrenalectomy; OR= odds ratio; LCI= Lower Confidence Interval; UCI=Upper Confidence Interval; LPrI= Lower predictive Interval; UPrI=Upper predictive Interval.

**Supplementary Figure 5- Panel A, mortality and morbidity**

**
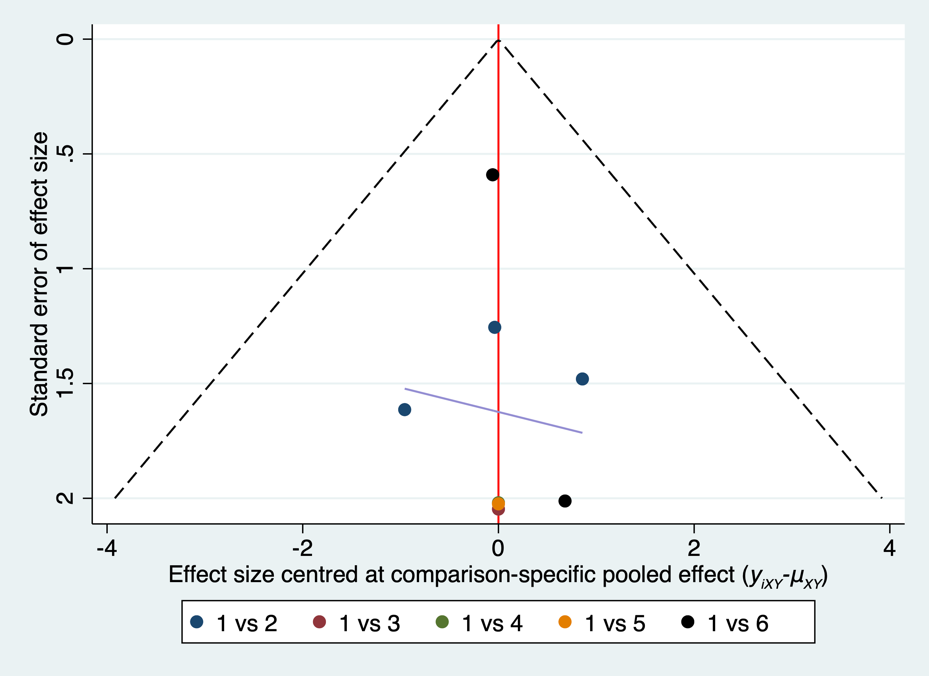
**

**Legend:** 1=Transperitoneal lateral laparoscopic approach; 2=Retroperitoneal lateral approach; 3=Robotic transperitoneal lateral approach; 4=Transperitoneal anterior laparoscopic approach; 5= Single-incision laparoscopic approach; 6= Retroperitoneal posterior approach.

**Supplementary Figure 5- Panel B, operative time**

**
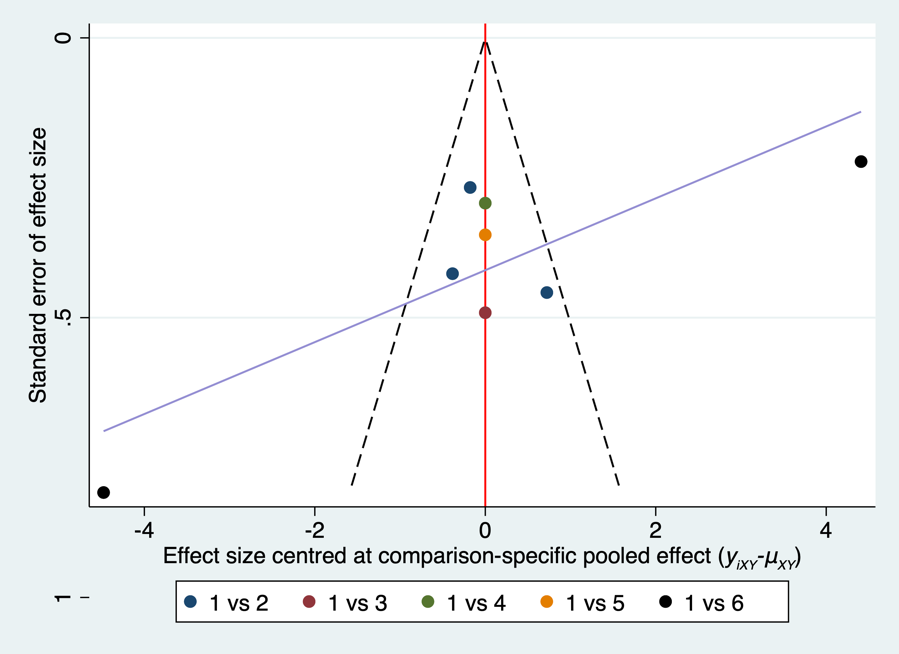
**

**Legend:** 1=Transperitoneal lateral laparoscopic approach; 2=Retroperitoneal lateral approach; 3=Robotic transperitoneal lateral approach; 4=Transperitoneal anterior laparoscopic approach; 5= Single-incision laparoscopic approach; 6= Retroperitoneal posterior approach.

**Supplementary Figure 5- Panel C, blood loss**

**
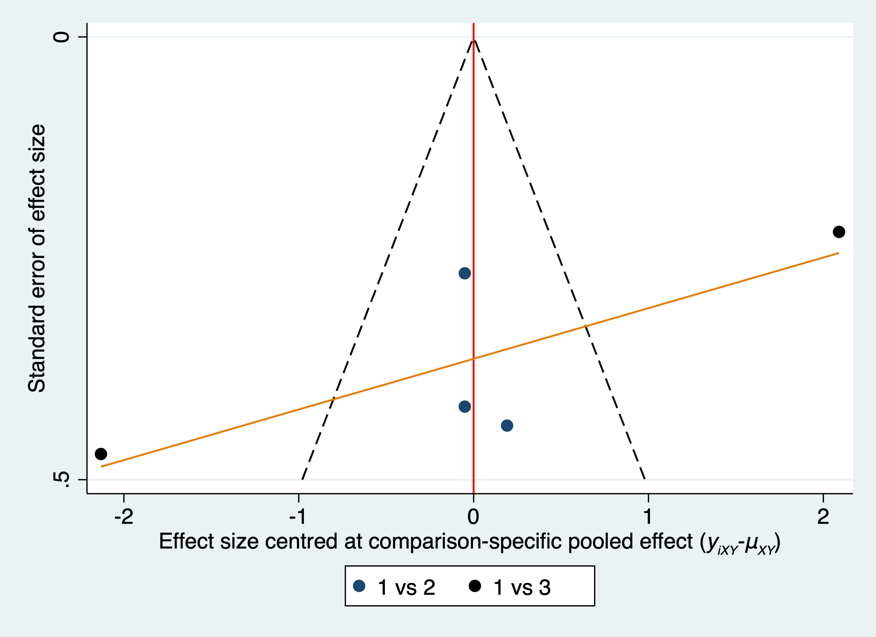
**

**Legend:** 1= Transperitoneal lateral laparoscopic approach; 2=Retroperitoneal lateral approach; 3= Retroperitoneal posterior approach.

**Supplementary Figure 5- Panel D, length of stay**

**
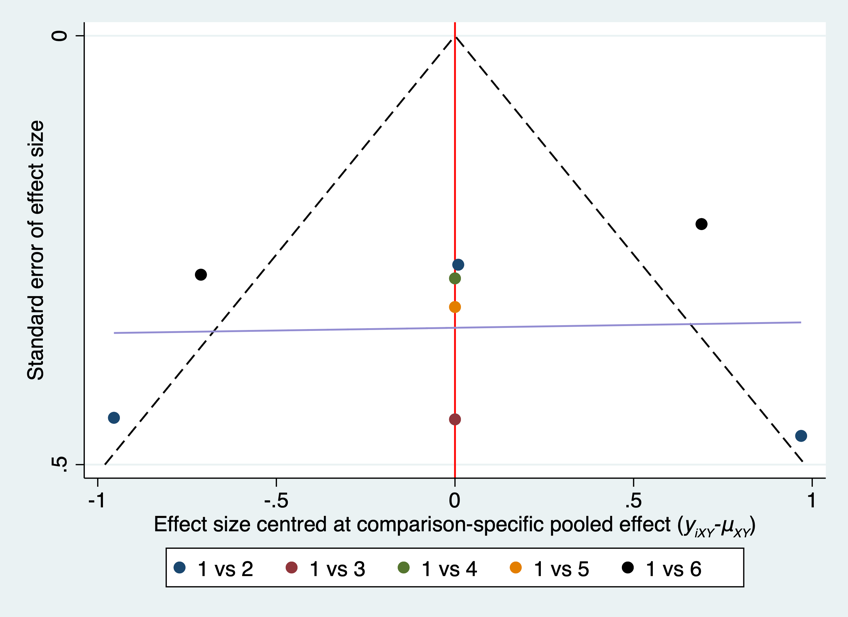
**

**Legend:** 1= Transperitoneal lateral laparoscopic approach; 2=Retroperitoneal lateral approach; 3=Robotic transperitoneal lateral approach; 4=Transperitoneal anterior laparoscopic approach; 5= Single-incision laparoscopic approach; 6= Retroperitoneal posterior approach.

**Supplementary Figure 5- Panel E, Conversion**

**
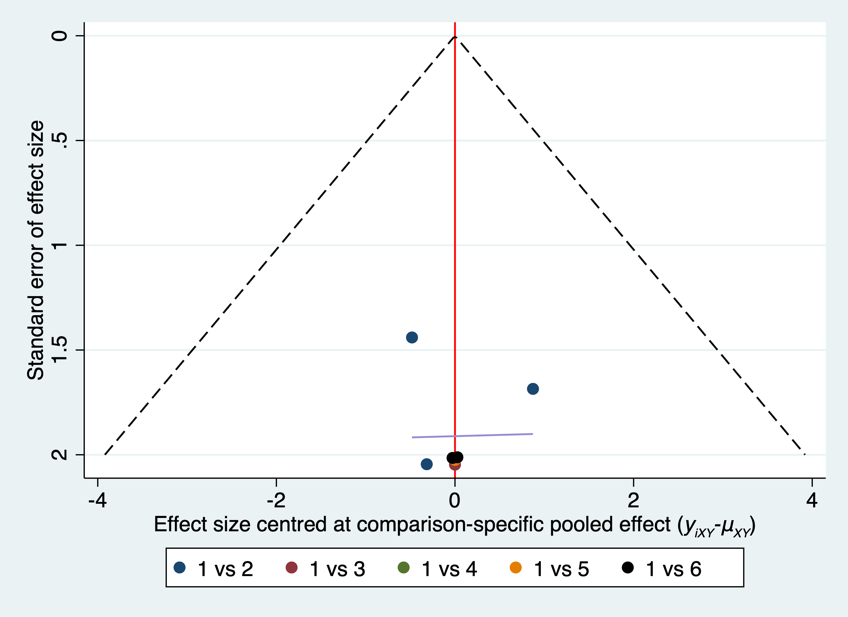
**

**Legend:** 1= Transperitoneal lateral laparoscopic approach; 2=Retroperitoneal lateral approach; 3=Robotic transperitoneal lateral approach; 4=Transperitoneal anterior laparoscopic approach; 5= Single-incision laparoscopic approach; 6= Retroperitoneal posterior approach.

**Supplementary Figure 5- Panel F, Incisional hernia**

**
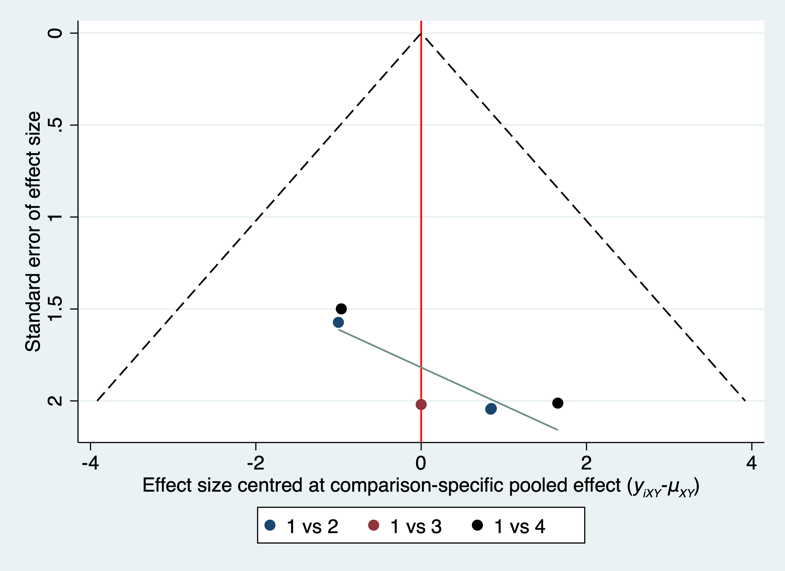
**

**Legend:** 1= Transperitoneal lateral laparoscopic approach; 2=Retroperitoneal lateral approach; 3=Transperitoneal anterior laparoscopic approach; 4= Retroperitoneal posterior approach.

**Supplementary Figure 5- Panel G, Disease recurrence**

**
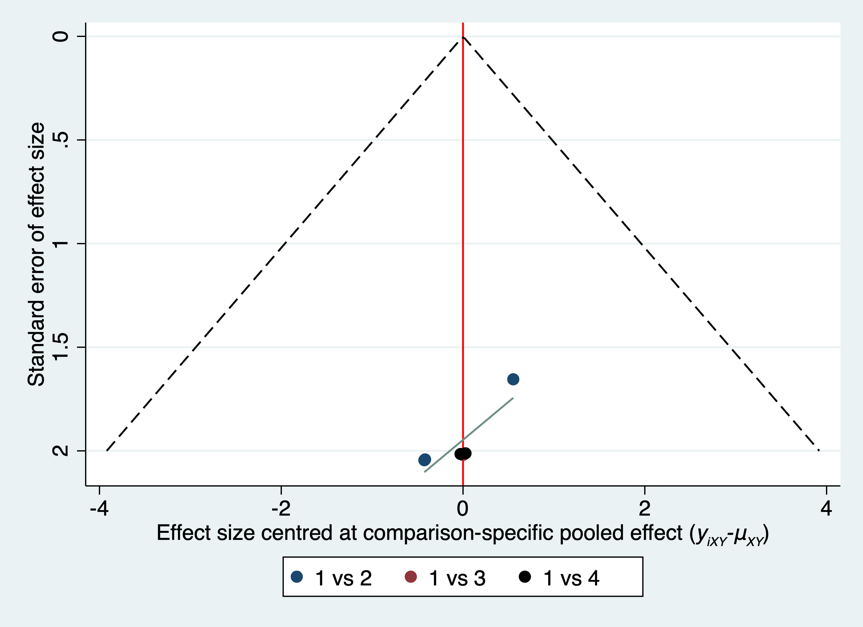
**

**Legend:** 1= Transperitoneal lateral laparoscopic approach; 2=Retroperitoneal lateral approach; 3=Transperitoneal anterior laparoscopic approach; 4= Retroperitoneal posterior approach.

**References**

1. Sterne JAC, Savović J, Page MJ, et al. RoB 2: a revised tool for assessing risk of bias in randomised trials. BMJ (in press)
2. Higgins JPT, Green S. Cochrane Handbook for Systematic Reviews of Interventions Version 5.1.0 [updated March 2011]. The Cochrane Collaboration, 2011. Available from http://handbook.cochrane.org. Accessed January 3, 2019.
3. Chaimani A, Higgins JP, Mavridis D, Spyridonos P, Salanti G. Graphical tools for network meta-analysis in STATA. PLoS One. 2013; 8: e76654.Clarke M, Horton R Bringing: Lancet-Cochrane collaborate on systematic reviews. Lancet 2001: 357; 1728.
4. Bucher HC, Guyatt GH, Griffith LE, Walter SD.The results of direct and indirect treatment comparisons in meta-analysis of randomised controlled trials. J Clin Epidemiol 1997; 50:683-691
5. Salanti G, Ades AE, Ioannidis JP. Graphical methods and numerical summaries for presenting results from multiple-treatment meta-analysis: an overview and tutorial. J Clin Epidemiol. 2011; 64: 163–171.
6. Bucher HC, Guyatt GH, Griffith LE, Walter SD. The results of direct and indirect treatment comparisons in meta-analysis of randomised controlled trials. J Clin Epidemiol 1997; 50:683-691
7. Shim S, Yoon BH, Shin IS, Bae JM.Network meta-analysis: application and practice using Stata. Epidemiol Health. 2017; 27;39:e2017047
8. Turner RM, Davey J, Clarke MJ, Thompson SG, Higgins JP. Predicting the extent of heterogeneity in meta-analysis, using empirical data from the Cochrane Database of Systematic Reviews. Int J Epidemiol. 2012;41: 818-827 .
9. Higgins JP, Thompson SG. Controlling the risk from spurious findings from meta-regression. Statistics in Medicine 2004; 23:1663-1682.
10. Manly BFJ. 2006. Randomization, Bootstrap and Monte Carlo Methods in Biology. 3rd ed. Boca Raton, FL: Chapman & Hall/CRC.
11. Egger M, Davey Smith G, Schneider M, Minder C. Bias in meta-analysis detected by a simple, graphical test. BMJ 1997: 315; 629-634
